# Correlation based feature importance analysis for improving machine learning stability predictions in hybrid PV systems

**DOI:** 10.1038/s41598-026-37270-y

**Published:** 2026-02-20

**Authors:** Veenita Swarnkar, Shimpy Ralhan, Mahesh Singh, Deepak Parashar, Mangal Singh

**Affiliations:** 1 Shri Shankaracharya Technical Campus, Bhilai, Chhattisgarh India; 2 Department of Robotics and Automation, Symbiosis Institute of Technology, Pune Campus, Symbiosis International (Deemed University), Pune, India; 3 Manipal Institute of Technology, Manipal Academy of Higher Education, Manipal, India; 4 Department of E&TC, Symbiosis Institute of Technology, Pune Campus, Symbiosis International (Deemed University), Pune, India

**Keywords:** Grid voltage forecasting, Gradient boosting, Hybrid photovoltaic systems, Feature importance, Grid stability enhancement, Renewable energy integration, Machine learning, Energy science and technology, Engineering, Mathematics and computing

## Abstract

Accurate prediction of grid voltage and stability is critical for ensuring reliable and efficient operation of modern power systems, especially with the increasing integration of intermittent renewable energy sources. This study rigorously evaluates five Machine Learning (ML) models, viz., Random Forest (RF), Extra Trees (ET), Support Vector Regression (SVR), Cat Boost (CB), and Gradient Boosting (GB), for their predictive performance in grid connected hybrid PV systems. Using a multimetric framework (R^2^, MAE, RMSE, MAPE) and advanced visual diagnostics (error distributions, temporal trend analysis), Gradient Boosting emerged as the top performing model, demonstrating superior accuracy and robustness across both voltage and stability prediction tasks. For grid voltage prediction, GB achieved the highest test R^2^ = 0.9785 and lowest MAPE = 0.25%, with 95% of errors confined to ± 0.5 V. In stability score forecasting, GB again outperformed all alternatives, attaining the best R^2^ = 0.9300 and lowest MAE = 0.75, while maintaining tight residual distributions ± 2.5 units. Comparative analysis revealed GB’s consistent superiority over tree based (RF, ET, CB) and kernel based (SVR) models, particularly in handling extreme operational ranges and temporal fluctuations. The results position Gradient Boosting as a unified, high precision solution for smart grid forecasting, offering actionable insights for real time monitoring and control. Its balanced performance across static and dynamic conditions underscores its suitability for resilient grid management in renewable rich environments. This work is novel in generating a controlled MATLAB/ Simulink dataset to capture nonlinear hybrid PV operating regimes and applying correlation-weighted feature engineering to enhance model interpretability. A unified benchmarking of five ML models under identical preprocessing identifies Gradient Boosting as the most reliable predictor. The framework further integrates extended KPIs and dynamic learning-rate scheduling, offering a robust and transparent approach for voltage and stability forecasting. Future research ought to examine hybrid GB ensembles and include optimization to enhance further on scalability with largescale deployments.

## Introduction

The electric power grid is undergoing a paradigm shift to intelligent and digitally controlled systems rather than the conventional electromechanical systems. The advanced grid infrastructures are intended to have advanced sensing, communication, information management and control systems, which integrate to constitute real time monitoring, databased decision making and increased operational efficiency^1^. The forward development of modern power systems and, especially the increased use of renewable energy sources, has added to the focus on intelligent computational methods of stability prediction, forecasting, and optimization of power systems. Situational awareness frameworks based on data have proven to have a great potential in improving smart-grid monitoring and operational decision-making processes by utilizing high dimensional measurements of the system and applying machine learning methods^2^. In addition to this, wide ranging reviews on solar energy modeling have noted the importance of proper irradiance prediction, hybrid system modeling and component level optimization to enhance the reliability of renewable integration^3^. The relevance of multi-energy system coordination optimization can also be demonstrated in more complicated micro grid work involving PV, wind, fuel cells and batteries where hybrid controls methods (Genetic Algorithms and Model Predictive Control) have a great impact on effectiveness^4^.

In this regard, machine learning predictive models offer a viable avenue towards enhancing predictability of voltage and stability in renewable integrated grids^5^. The proposed study incorporates correlation-based feature importance analysis, which means that the most influential variables influencing grid behavior are identified and model interpretability is improved. The overall objective of this integration is to enhance grid resilience and operational reliability in hybrid PVs to achieve data driven adaptability in the face of changing renewable conditions.

**Fig. 1:**
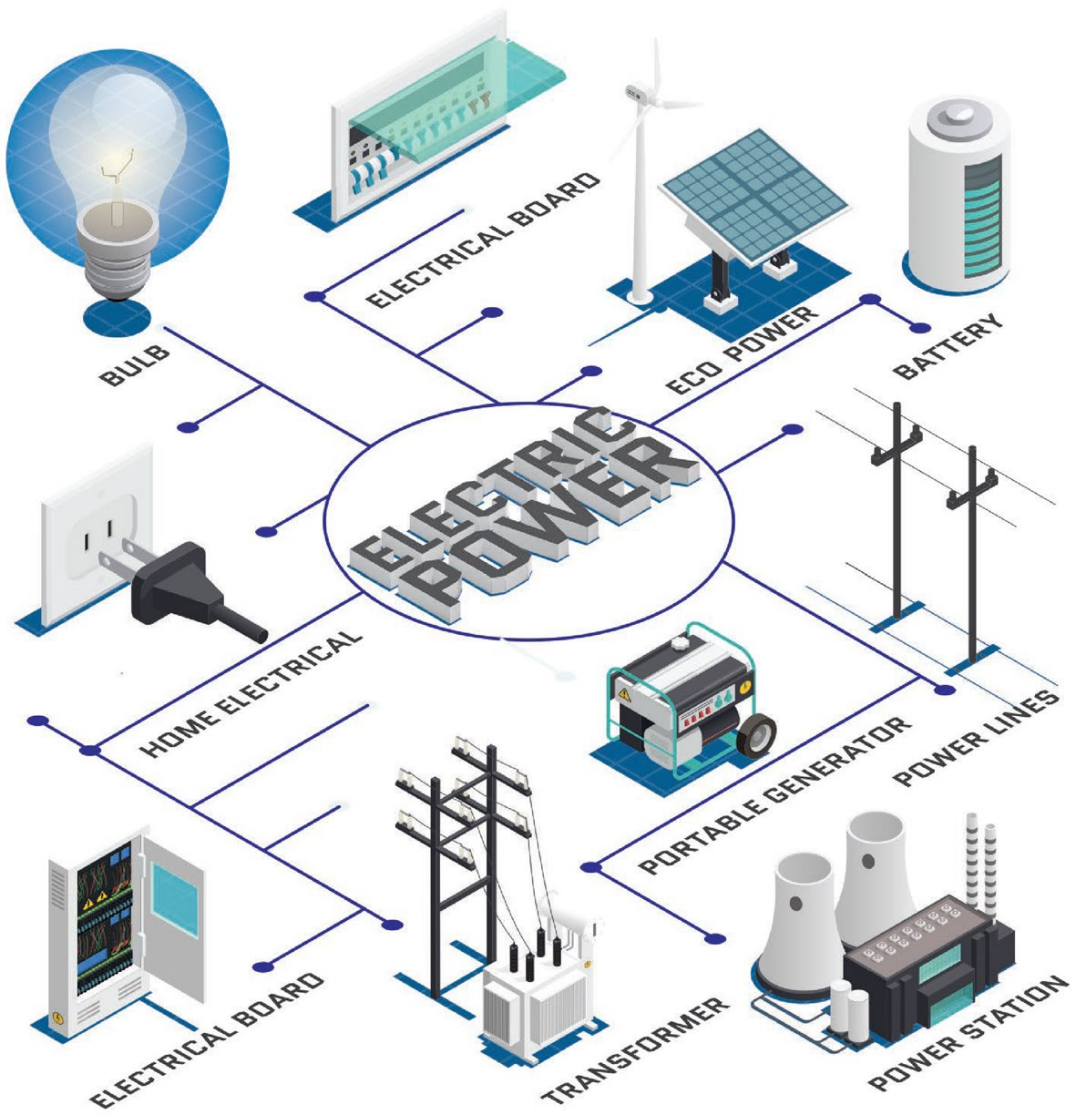
Integration of renewable and conventional sources in the grid.

Figure 1 depicts a typical system of electrical power that integrates both traditional and renewable energy sources or power. ML based models have been popular in the field of stability assessment because they can provide an approximation of the behavior of nonlinear systems more effectively than classical analytical models^6^. The use of ML models in power system stability assessment has been investigated recently, which provided information about the model modes and future research directions^7^. Deep learning methods, especially LSTM based models, have shown high performance when it comes to short term voltage stability prediction with post disturbance trajectories^8^ whereas complementary strategies use local regression to predict adaptively voltage-stability margins in real time^9^. ML and 1D CNN based fault detection schemes have demonstrated strong performance in detecting abnormal conditions in grid connected PV systems whose performance has been verified in simulation and real time experimental results^10^. A detailed analysis of AI methods also highlights how CNNs, ensemble learning, XG Boost, and hybrid feature selection approaches are increasingly used to solve various problems of a smart grid (i.e., fault detection, theft prevention, and renewable forecasting)^11^. The investigations in hybrid renewable energy systems also manifest the fact that parallel fusion methods were used, which involved deep learning and hybrid ML models to optimize the work of PV wind better^12^. Oda et al.^13^ addresses the allocation of a hybrid system that includes PV-DG and DSTATCOM. The planning problem considers the variations of load demand and solar irradiance under deterministic and probabilistic conditions.

Forecasting has continued to be a foundation of hybrid renewable management. Researchers have suggested Adaptive ML based models in prediction of renewable power in PV wind hybrid farms, which show better accuracy in varying environmental conditions^14^. Hybrid machine learning forecasting models of PV power have add on to the advancement of the field with integrated model of complex relationships of irradiance and power^15^. It has been demonstrated through grid integration works that intelligent virtual inertia synthesis through synchronverters can greatly improve PV system support during disturbances events^16^, and that hybrid PV wind system integration structures can be developed to reduce uncertainty and improve grid reliability^17^. Other contributions include optimization of grid connected hybrid systems with multiple objectives in mind, with an emphasis on cost, reliability and environmental performance^18^. Smart controls of hybrid wind PV systems remain at better voltage regulation and coordinated control of power at variable flows of renewables^19,20^.

In addition to the power system use, data driven prediction and performance analysis techniques have also become a useful tool in industrial IoT ecosystems, which supports the wider applicability of ML based prediction tools^21^. Early research on solar power generation has also highlighted the need to have correct PV modeling, irradiance prognostication and system level efficiency factors^22^. Smart operation of wind PV hybrid systems has been steadily developed and has offered necessary informational perspective in inverter design, MPPT approaches, and hybrid planning^19^. In the meantime, hybrid ML and feature selection approaches have been implemented successfully to predict the energy production in PV wind renewable systems, which underscores the value of dimensionality reduction in model performance^23^.

More complex ML methods have also been proposed to control converters (including predictor-based data driven adaptive control that can be used to enhance transient response in power converters^24^ and algorithms like R^2^ have been demonstrated to be more useful than traditional regression measures like MAPE and RMSE in assessing the accuracy of ML models^25^. Although both used predictor based adaptive control and statistical measures of evaluation, respectively, our study builds upon those studies by adding the concept of correlation based feature weighting as part of a Gradient Boosting system in order to enhance predictability of stability as well as explaining it via interpretability. This combination fills the divide between simple statistical evaluation and smart learning-based prediction, providing a holistic system of data-driven analysis of hybrid PV systems. One more application of generative Adversarial Networks (GANs) to dynamic security assessment has been offered where the missing data are adequately dealt with and realistic system states are generated^26^. Hybrid boosting models like Light GBM XG Boost ensembles have been shown to have high predictive power in regression challenges^27^, although transfer and ensemble learning have been applied in cross-domain tasks (sentiment analysis) where they show the flexibility of ML approaches^28^. The study of fundamental error analysis has also enhanced the knowledge of the behavior of Mean Squared Error (MSE) that is utilized in enhanced assessment of ML regression models^29^.

In more recent times, the use of fractional order controllers with repetition neural architecture has been implemented into PV systems providing enormous improvements in the performance of power quality^30^. The studies on PV systems in extreme weather environments will offer valuable information on the system susceptibility, adaptive control, and mitigation routes that are key to resiliency planning^31^. Further improvement in the methodological space includes advanced architectures of PV fault detection, deep ensemble forecasting, hybrid renewable optimization, physics informed ML and AI enhanced inverter control with applications including hybrid PV wind forecasting, reactive power support, stability prediction and adaptive control under uncertain operation conditions^11,32–43^.

Though these are quite important contributions, a number of gaps still exist. Research on this area is usually confined to specific activities like forecasting, fault detection, inverter control or uncertainty modeling and not offer an integrated framework that brings together feature importance analysis, multi-model benchmarking, correlation-weighted feature engineering and prolonged Key Performance Indicator (KPI) evaluation. Sensitivity analysis, learning rate adaptation, and interpretable feature weighting is almost never used in existing work, which is important in practice (especially grid operations) and operator confidence. This loophole inspires the current research where it is proposed to create a correlation-weighted machine learning model that improves predictive quality, model interpretability, and reliability in the assessment of stability in hybrid PV systems.

As noted in the literature review Table 1, the problems that were found to be performed by the current studies are mainly based on PV forecasting, inverter control, or statistical evaluations of grid performance, but none of them include correlation driven feature weighting to be more effective in stability prediction. None of the reviewed publications discusses at the same time extended KPIs, dynamic learning rate scheduling, sensitivity analysis and multimodal performance benchmarking. This limitation inspires the current paper, which incorporates correlation weighted feature importance with several ML regressors to offer a more valid and presentable stability prediction framework of hybrid PV systems.

**Table 1:** Comparison table of literature review.

Ref	Study focus	Dataset size	Features considered	ML models used	Stability metric	Feature engineering	Novelty claim	Limitations
^21^	PV power forecasting with ML	Medium	Irradiance, temperature	RF, SVR	RMSE	Minimal	Model accuracy improvement	No stability analysis
^22^	Grid connected PV voltage estimation	Small	V_grid_, I_grid_, PV power	ANN	MAPE	None	Incremental	No feature weighting; limited dataset
^19^	Hybrid PV–wind system behavior	Large	Multiple environmental inputs	GBM	MAE	None	Multisource modeling	No sensitivity analysis
^51^	Voltage stability in distribution systems	Medium	Voltage, load, impedance	SVM	VSI	Limited	Stability index prediction	Not ML based forecasting
^23^	Grid reliability under PV penetration	Medium	PV penetration %, load	Statistical models	Reliability index	None	Penetration modelling	No Realtime prediction
^24^	ML for inverter control	Small	Switching signals	DL models	Regulation error	Basic scaling	Advanced DL	No system level stability
^25^	Voltage prediction in smart grids	Medium	Voltage, load	X G Boost	RMSE	None	High-performance model	No correlation-based weighting
^26^	PV stability forecasting with DL	Medium	PV power, voltage	CNN and LSTM	MAE	Sequence features	Temporal modelling	Limited explain ability
^27^	Renewable forecasting review	–	–	Various	–	–	Comprehensive survey	No original experiment
^28^	Solar irradiance forecasting	Medium	GHI, DNI	Cat Boost	RMSE	None	Model level innovation	No grid stability
OurWork	Hybrid PV stability prediction with correlation weighted ML	500 × 6	Irradiance, temp, load, DC link V, inverter P, grid I	RF, ET, SVR, CB, GB	RMSE, MAPE, MARE, RMSRE, RMSPE	Correlation based feature weighting	Novel multimodal comparison + weighted GB	Addresses gaps: feature influence, extended KPIs, LR decay, sensitivity

This work advances smart grid forecasting through:

1. A MATLAB simulation of the Hybrid PV inverter grid system is used to generate the complete dataset, which enables the control of operating conditions and dynamic behavior of the power system.
2. Introduction of correlation-based feature analysis to enhance model interpretability.
3. Identification of Gradient Boosting as a robust single model solution for both voltage and stability prediction.
4. Use of advanced diagnostics, including residual and error distribution analysis, for deeper performance validation.
5. Practical insights supporting reliable, real time grid monitoring in renewable integrated environments.

The parts of this work are divided into the following: “System modeling” section describes system modeling on the evaluation of voltage stability concerning Inverter Based Resources (IBRs). The 3rd section describes the suggested method of estimating voltage stability, with the help of an SVM based system model within the power system. “Results and discussion” section provides explanation and commentary to the findings and “Practical implications” section ends the article by reviewing the findings and suggestions to the future research.

The major research gaps addressed in this work are summarized as follows:

1. Lack of model interpretability in existing AI based stability assessment studies, which limits their practical use in operational grids.
2. Limited comparative evaluation of multiple machine learning regressors under identical experimental conditions for hybrid PV systems.
3. Absence of correlation weighted feature engineering strategies in previous PV voltage stability prediction frameworks, leading to suboptimal learning efficiency.
4. Insufficient integration of statistical analysis and ML based feature importance, restricting explain ability and predictive reliability for smart grid applications.

The novelty of this study lies in integrating correlation-based feature weighting with ensemble machine learning regression to enhance both the accuracy and interpretability of stability prediction in hybrid photovoltaic systems.

This study introduces a correlation-weighted machine learning framework for hybrid PV voltage and stability prediction, supported by a systematically generated dataset obtained from an algorithmic MATLAB/Simulink simulation environment. Unlike conventional data-driven studies that rely solely on field measurements, this work constructs a controlled and reproducible dataset by varying irradiance, temperature, load conditions, and inverter operating states within a validated MATLAB model. This ensures full coverage of nonlinear operating regimes and provides a reliable basis for training and evaluating the proposed machine learning models.

The proposed framework incorporates correlation-driven feature weighting to highlight the most influential physical variables, thereby improving model convergence and interpretability. A unified benchmarking of five machine learning algorithms, viz., Random Forest, Extra Trees, SVR, CatBoost, and Gradient Boosting is conducted under identical preprocessing, normalization, and evaluation pipelines to ensure fair and unbiased comparison. To achieve deeper reliability assessment, the study adopts an extended KPI suite (MARE, RMSPE, RMSRE) in addition to conventional metrics such as R^2^, MAE, RMSE, and MAPE.

A dynamic learning rate decay mechanism is embedded within the Gradient Boosting model to enhance training stability and reduce overfitting relative to fixed learning rate implementations. Furthermore, the framework integrates correlation analysis, feature weighting, and sensitivity evaluation into a coherent interpretability pipeline, offering a transparent, physics-consistent methodology for forecasting voltage and stability in hybrid PV systems based on algorithmically generated simulation data.

## System modeling

The microgrid is modeled as an integrated system comprising renewable energy generation, consumer loads, and the grid interface through power electronics. The dynamic behavior of each subsystem is described using mathematical formulations, while a data driven regression framework is employed for performance prediction^17^. We model a grid connected PV inverter load microgrid with well-posed device equations and a supervised learning map that predicts PCC/Grid voltage and a dimensionless stability score. Unless stated otherwise, all variables are in SI units, steady state phasors have RMS magnitudes, and bold symbols denote vectors. We have made the assumptions that there is balanced three phase operation; fundamental frequency dynamics (harmonics filtered by LCL); small electrical angle excursions (|δ|≲15°) when linearized and parameters are constant over an identification interval.

The novelty of this study lies in developing a correlation-weighted ensemble learning framework for which the dataset has been generated in the MATLAB environment that integrates statistical dependence metrics with Gradient Boosting regression to enhance accuracy and interpretability in hybrid PV stability prediction. Unlike previous studies, the proposed model unifies feature importance quantification, dynamic learning rate scheduling, and extended KPI benchmarking, providing a reproducible pipeline for smart grid forecasting.

### Renewable energy generation (PV model)

The photovoltaic source is modeled using the well-established single-diode five-parameter representation^44^. For a PV module consisting of *N*_s_ series-connected cells, the terminal current–voltage relationship satisfies the nonlinear algebraic Eq. (1).

1 $$I_{pv} = I_{ph} - I_{0} \left[ {\exp \left( {\frac{{V_{pv} + I_{pv} R_{s} }}{{nV_{t} }}} \right) - 1} \right] - \frac{{V_{pv} + I_{pv} R_{s} }}{{R_{sh} }}$$

where $V_{pv}$ and $I_{pv}$ are the terminal voltage and current, $R_{s}$ and $R_{sh}$ are the series and shunt resistances, and *n* is the diode ideality factor. The thermal voltage ($V_{t}$) is defined in (2).

2 $$V_{t} = \frac{kT}{q}$$

with *k* the Boltzmann constant, *q* the electron charge, and *T* the cell temperature in Kelvin.

The photocurrent varies with irradiance and temperature according to (3).

3 $$I_{ph} = I_{sc} \left[ {1 + \alpha_{T} \left( {T - T_{ref} } \right)} \right]\frac{G}{{G_{ref} }}$$

where $I_{sc}$ is the reference short-circuit current, $\alpha_{T}$ is its temperature coefficient, and *G* and $G_{ref}$ are the instantaneous and reference irradiance levels. The diode saturation current is modeled as in (4).

4 $$I_{0} = I_{0,ref} \left( {\frac{T}{{T_{ref} }}} \right)\exp \left[ {\frac{{E_{g} }}{{nV_{t} }}\left( {\frac{1}{{T_{ref} }} - \frac{1}{T}} \right)} \right]$$

where $I_{0,ref}$ is the reference saturation current and $E_{g}$ is the semiconductor bandgap energy.

Equations (1) to (4) constitute a nonlinear current–voltage relationship that is monotonic in $I_{pv}$ for $R_{s} \ge 0$. The equation admits a unique physically valid solution for each operating point $\left( {V_{pv} ,G,T} \right)$, and the algebraic loop is solved numerically using Newton’s method. The Jacobian of the update map is locally Lipschitz on compact $\left( {V_{pv} ,I_{pv} } \right)$ domains, ensuring stable and convergent iteration.

The instantaneous PV output power is given by (5).

5 $$P_{pv} = V_{pv} \times I_{pv}$$

This model accurately captures the temperature dependent, irradiance dependent, and nonlinear electrical behavior of the PV source and is widely used in stability and control studies of hybrid renewable systems.

### Aggregated load (ZIP) model

The aggregated demand at the point of common coupling is represented using a voltage and frequency dependent ZIP load model. Let *V* denote the RMS voltage magnitude at the PCC and *f* the system frequency^45^. The real and reactive power consumptions are expressed as in (6) and (7).

6 $$P\left( {V,f} \right) = P_{0} \left[ {a_{p} \left( {\frac{V}{{V_{0} }}} \right)^{2} + b_{p} \left( {\frac{V}{{V_{0} }}} \right) + c_{p} } \right]\left( {1 + k_{p} \left( {f - f_{0} } \right)} \right)$$

7 $$Q\left( {V,f} \right) = Q_{0} \left[ {a_{q} \left( {\frac{V}{{V_{0} }}} \right)^{2} + b_{q} \left( {\frac{V}{{V_{0} }}} \right) + c_{q} } \right]\left( {1 + k_{q} \left( {f - f_{0} } \right)} \right)$$

where $P_{0}$ and $Q_{0}$ are the nominal active and reactive power demands at nominal operating conditions $\left( {V_{0} ,f_{0} } \right)$. The coefficients are given by (8) and define the constant impedance $\left( Z \right)$, constant current $\left( I \right)$, and constant power $\left( P \right)$ components of the aggregated load.

8 $$a_{p} + b_{p} + c_{p} = 1\,,\,\,\,\,\,\,\,\,\,\,\,\,a_{q} + b_{q} + c_{q} = 1$$

The terms $k_{p}$ and $k_{q}$ are small frequency sensitivity coefficients that capture the primary frequency dependence of the load. For completeness, the canonical ZIP polynomial representation (voltage dependency only) is given in (9) and (10).

9 $$P_{ZIP} = P_{0} \left[ {a_{p} \left( {\frac{V}{{V_{0} }}} \right)^{2} + b_{p} \left( {\frac{V}{{V_{0} }}} \right) + c_{p} } \right]$$

10 $$Q_{ZIP} = Q_{0} \left[ {a_{q} \left( {\frac{V}{{V_{0} }}} \right)^{2} + b_{q} \left( {\frac{V}{{V_{0} }}} \right) + c_{q} } \right]$$

Equations (6) and (7) extend (9) and (10) by incorporating explicit frequency sensitivity, enabling the load model to represent realistic operating variations in weak and hybrid renewable dominated grids.

The corresponding load currents injected at the PCC follow from (11).

11 $$I_{P} = \frac{{P\left( {V,f} \right)}}{V},\,\,\,\,\,\,\,I_{Q} = \frac{{Q\left( {V,f} \right)}}{V}$$

and the net complex load current is given by (12).

12 $$I_{load} = I_{P} - jI_{Q}$$

### Grid interface and inverter dynamics

The hybrid PV system interfaces with the grid through a current-controlled Voltage Source Converter (VSC) equipped with an L-filter^46^. The inner capacitor of an LC filter is neglected based on bandwidth separation. The converter is modeled in the synchronous $d_{q}$ reference frame aligned with the grid voltage vector. The filter dynamics are described by (13) and (14).

13 $$L\frac{{di_{d} }}{dt} = - Ri_{d} + \omega Li_{q} + v_{d} - v_{od}$$

14 $$L\frac{{di_{q} }}{dt} = - Ri_{q} + \omega Li_{d} + v_{q} - v_{oq}$$

where $i_{d}$, $i_{q}$ are the converter currents, $v_{d}$, $v_{q}$ are converter output voltages, $v_{od}$, $v_{oq}$ are PCC voltages, *L* and *R* represent filter inductance and resistance, and ω is the grid angular frequency.

The converter voltage is generated through PWM modulation of the DC-link voltage $V_{dc}$. For a modulation index $m \in \left( {0,1} \right)$, $v_{d}$ and $v_{q}$ are as given in (15) and (16).

15 $$v_{d} = \frac{{mV_{dc} }}{2}\cos \phi$$

16 $$v_{q} = \frac{{mV_{dc} }}{2}\sin \phi$$

where *ϕ* is the modulation angle.

The instantaneous active and reactive powers injected at the PCC are expressed in the grid aligned $d_{q}$ frame as in (17).

17 $$P = \frac{3}{2}v_{d} i_{g,d} ,\,\,\,\,\,\,\,\,\,\,Q = - \frac{3}{2}v_{d} i_{g,q}$$

If the coupling is predominantly inductive $\left( {X > > R} \right)$ and the inverter internal EMF is *E* behind reactance *X*, the classical power transfer relations are given in (18).

18 $$P \approx \frac{{EV_{g} }}{X}\sin \delta ,\,\,\,\,\,\,\,\,Q \approx \frac{E}{X}\left( {E - V_{g} \cos \delta } \right)\,\,$$

valid for small torque angle $\left| \delta \right|$ and $R/X < < 1$. These are used only for set point selection; closed loop simulations use (13) and (17).

A decoupled PI current controller regulates the $d_{q}$ currents according to (19) and (20).

19 $$v_{d}^{*} = v_{od} - \omega Li_{q} + K_{pd} \left( {i_{d}^{*} - i_{d} } \right) + K_{id} \int {\left( {i_{d}^{*} - i_{d} } \right)dt}$$

20 $$v_{q}^{*} = v_{oq} - \omega Li_{d} + K_{pq} \left( {i_{q}^{*} - i_{q} } \right) + K_{iq} \int {\left( {i_{q}^{*} - i_{q} } \right)dt}$$

where $K_{pd}$, $K_{id}$, $K_{pq}$ and $K_{iq}$ are positive controller gains ensuring exponential convergence in the tracking error.

Equation (21) gives the DC-link dynamic equation governing the energy balance of the converter.

21 $$C\frac{{dV_{dc} }}{dt} = i_{pv} - \frac{3}{2}\left( {v_{d} i_{d} + v_{q} i_{q} } \right)$$

where *C* is the DC-link capacitor and $i_{pv}$ is the PV array current feeding the DC-bus. Equation (21) ensures conservation of power between the PV generator, converter switching actions, and AC output power.

### Differential algebraic model and PCC power balance

The hybrid PV inverter grid system is expressed as a set of Differentials Algebraic Equations (DAEs) in (22)^47^.

22 $$\dot{x} = f\left( {x,y,u} \right),\,\,\,\,\,\,\,\,\,\,\,0 = g\left( {x,y,u} \right)$$

where *x* collects the dynamic states (e.g., inverter currents $i_{d} ,i_{q} ,$ DC-link voltage $V_{dc}$, PV internal states), *y* contains algebraic variables (e.g., PCC voltages $v_{od} , \, v_{oq}$, voltage magnitude *V*, angle *θ*), *u* denotes external inputs (irradiance *G*, temperature *T*, nominal load powers $P_{0} ,Q_{0}$, grid frequency *f*, current references $i_{d}^{ * } ,i_{q}^{ * }$).

The differential part $f( \cdot )$ is determined by the PV model, inverter filter and DC link dynamics (Eq. 1–5 and 6–12), while $g( \cdot )$ enforces algebraic network constraints at the PCC. In particular, the active and reactive power injections from the inverter must balance the ZIP load and line losses. Let (*P*,* Q*) be the inverter powers from (9), $\left( {P_{\ell } ,Q_{\ell } } \right)$ the ZIP load powers from (4) and (5), and $R_{\ell } ,X_{\ell }$ the equivalent line resistance and reactance.

With $I = \sqrt {i_{d}^{2} + i_{d}^{2} }$, the RMS PCC current, the power balance constraints are given by (23).

23 $$P = P_{\ell } - R_{\ell } I^{2} = 0,\,\,\,\,\,\,\,\,\,\,\,\,Q = Q_{\ell } - X_{\ell } I^{2} = 0$$

which are included in the algebraic part $g\left( {x,y,u} \right) = 0$. The DAE formulation is used as the backbone for time domain simulations and for generating the dataset employed in the subsequent machine learning based stability prediction.

### Stability metrics

To quantitatively assess the operating condition of the hybrid PV inverter grid system^48^, two complementary stability indicators are defined as voltage deviation index ${{\boldsymbol{\upzeta}}}$ and bounded composite stability score *S***.**

- Voltage deviation index

The average normalized deviation of the PCC voltage from its nominal value $V_{ref} = 1$ over an evaluation window of *N* samples is expressed in (24).

24 $$\zeta = \frac{1}{N}\sum\limits_{k = 1}^{N} {\left| {\frac{{V_{g} \left( k \right) - V_{ref} }}{{V_{ref} }}} \right|}$$

A lower value of ${{\boldsymbol{\upzeta}}}$ indicates improved voltage regulation and disturbance resilience.

(b) Composite Stability Score

To capture additional dynamic characteristics of frequency deviation, damping behavior, and controller saturation, the bounded stability score is defined in (25).

25 $$S = \frac{1}{4}\left( {S_{v} + S_{f} + S_{\xi } + S_{sat} } \right)$$

with each sub-component normalized to the interval [0,1], ensuring $0 \le S \le 1$.

The voltage and frequency performance terms are given in (26).

26 $$S_{v} = 1 - \frac{1}{N}\sum\limits_{k = 1}^{N} {\left| {\frac{{V_{g} \left( k \right) - V_{ref} }}{{V_{ref} }}} \right|} ,\,\,\,\,\,\,\,\,S_{f} = 1 - \frac{1}{N}\sum\limits_{k = 1}^{N} {\left| {\frac{{f\left( k \right) - f_{ref} }}{{f_{ref} }}} \right|} \,\,$$

The damping quality term is derived from the minimum damping ratio $\xi_{min}$ of the dominant linearized poles as in (27).

27 $$S_{\xi } = \min \left( {1,\frac{{\xi_{\min } }}{{\xi_{ref} }}} \right)$$

where$\xi_{ref}$ denotes the reference desired damping ratio

The control saturation penalty ($S_{sat}$) is defined by (28).

28 $$S_{sat} = 1 - \max \left( {\frac{{m\left( k \right) - m_{\min } }}{{m_{\max } - m_{\min } }}} \right)$$

where $m\left( k \right)$ is the modulation index and $m_{\max } = 1$ denotes full modulation.

The index *ζ* captures average voltage deviation, whereas *S* aggregates four normalized indicators to provide a bounded measure of overall stability. The mapping is calibrated so that $S = 1$ corresponds to well-damped, unsaturated operation with negligible voltage and frequency deviations. Both *ζ* and *S* serve as supervised learning targets in the ML based stability prediction framework.

### Stability prediction framework

This work employs supervised learning to predict voltage and dynamic stability outcomes of the hybrid PV inverter grid system. Two complementary stability indicators are defined from time domain simulations of the DAE model. The first indicator measures average normalized PCC voltage deviation over an evaluation window of length N as given in (24). To capture additional characteristics of frequency deviation, damping, and control saturation, a bounded stability score S ∈ [0,1] is defined as in (25).

Time-domain simulations of the DAE system generate a dataset as expressed in (29).

29 $$D\mathop = \limits^{\Delta } \left\{ {\left( {x_{i} ,y_{i} } \right)} \right\}_{i = 1}^{N}$$

where each feature vector $x_{i}$ contains environmental and operational covariates (irradiance, temperature, load levels, inverter states), standardized to zero mean and unit variance. The targets are given in (30).

30 $$y_{i} = \left( {\zeta_{i} ,S_{i} } \right)$$

representing instantaneous and composite stability measures.

A parametric regressor $f\theta ( \cdot )$ (e.g., Gradient Boosting, CatBoost, SVR, or neural networks) predicts stability outcomes as in (31).

31 $$\hat{y}_{i} = f_{\theta } \left( {x_{i} } \right)$$

We train by minimizing a weighted, dimensionally consistent objective with Tikhonov regularization as shown in (32).

32 $$\hat{\theta } = \arg \min \frac{1}{N}\sum\limits_{i = 1}^{N} {\left( {\omega_{\zeta } L_{\zeta } \left( {\zeta_{i} ,\hat{\zeta }_{i} } \right) + \omega_{S} L_{S} \left( {S_{i} ,\hat{S}_{i} } \right)} \right)} + \lambda \left\| \theta \right\|_{2}^{2}$$

where $\omega_{\zeta }$ and $\omega_{S}$ are inverse empirical variances ensuring scale balance, and λ is the regularization weight.

For probabilistic models, the regressor outputs a Gaussian mean–variance pair $\left( {\mu_{i} ,\sigma_{i}^{2} } \right)$, optimized via the negative log-likelihood as in (33).

33 $$L_{NLL} = \frac{1}{2}\left[ {\log \sigma_{i}^{2} + \frac{{\left( {y_{i} - \mu_{i} } \right)^{2} }}{{\sigma_{i}^{2} }}} \right]$$

allowing the model to learn aleatoric uncertainty.

The integrated framework combines physically grounded stability metrics with a mathematically well-posed supervised learning formulation. This enables consistent prediction of both voltage deviation and overall dynamic stability from environmental and operational features, supporting data-driven control and monitoring of hybrid PV systems.

The system model in “Renewable energy generation (PV model)”–“Stability metrics” section is developed under standard assumptions to ensure mathematical tractability and consistent dynamic behavior. The assumptions are that the PV module temperature is uniform, inverter switching dynamics are represented using an averaged model, the grid is assumed balanced and sinusoidal, enabling a synchronous $d_{q}$ reference frame, ZIP load coefficients satisfy the normalization conditions $a_{p} + b_{p} + c_{p} = 1$ and $a_{q} + b_{q} + c_{q} = 1$ and environmental variables (irradiance G and temperature T) vary slowly relative to inverter electrical dynamics.

### Conceptual map of the proposed framework

**Fig. 2:**
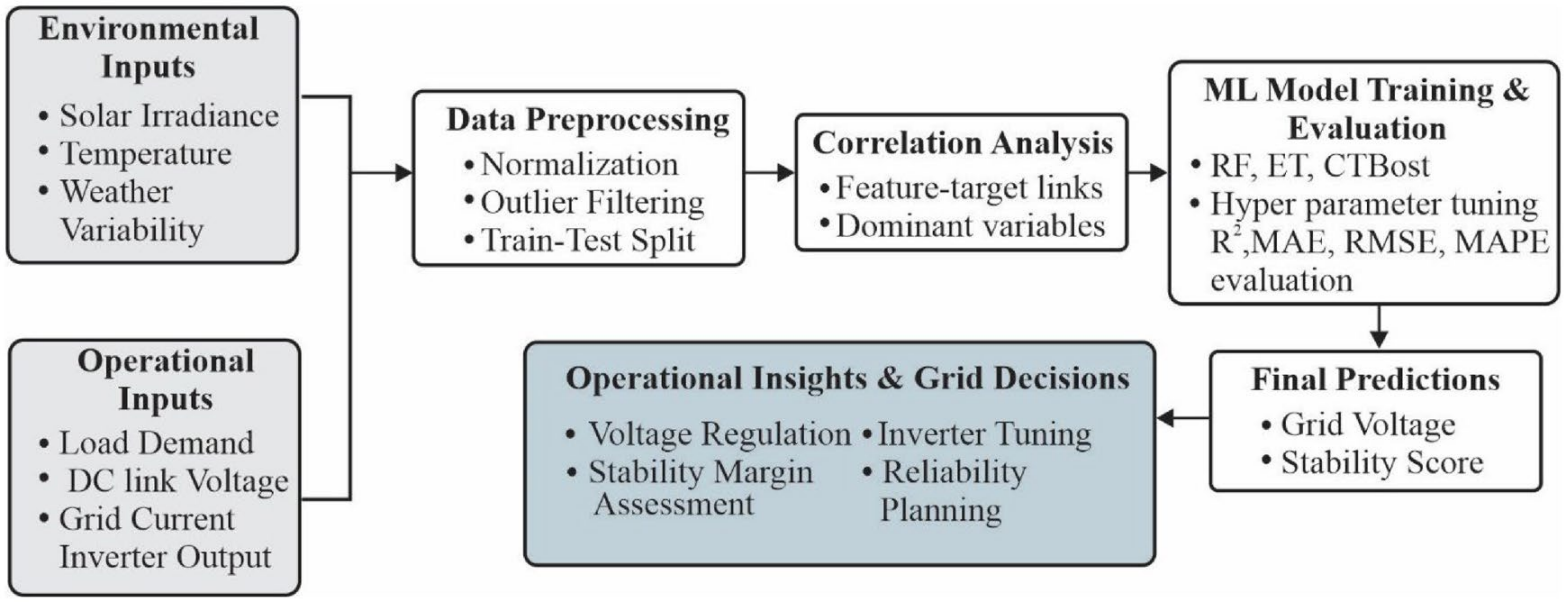
Conceptual map of the proposed correlation weighted ML framework.

Figure 2 illustrates the conceptual map of the proposed correlation weighted machine learning framework. The process begins with environmental and operational inputs obtained from the hybrid PV system, including temperature, irradiance, load demand, DC bus voltage, grid current, and inverter output power^49^. These raw inputs form the basis for both deterministic modelling and data driven learning. The next stage involves feature preprocessing and correlation analysis, where each variable’s statistical influence on grid voltage and stability is quantified. Correlation derived feature weights are then assigned to highlight the most dominant physical parameters.

Subsequently, the weighted features are fed into a suite of machine learning regressors (RF, ET, SVR, CB, GB), where Gradient Boosting is identified as the optimal model based on multimetric evaluation (R^2^, MAE, RMSE, MAPE). The model outputs two key predictions, i.e., grid voltage and stability score. These predictions are further validated through sensitivity analysis, feature importance assessment, and extended KPIs such as MARE, RMSRE, and RMSPE. Finally, the predicted quantities support operational decision making in grid voltage regulation, inverter control, and stability management under varying renewable conditions.

## Methodology used for stability prediction

The suggested methodology is aimed to solve the major issues of dynamic performance enhancement and power quality improvement in a grid connected hybrid PV Wind system. The hybrid system tends to have oscillating power production, voltage distortion, frequency variations and harmonic distortion because of intermittency and stochasticity of solar irradiance and wind velocity. Besides affecting the reliability of power supply, these challenges also make it difficult to integrate renewable energy into the grid smoothly^50^.

To address these constraints, the methodology uses smart computational methods, which are very important in ensuring maximal use of renewable. Soft computing Maximum Power Point Tracking (MPPT) algorithms are implemented to maintain the highest energy harvesting at any given environmental state, and the Flexible AC Transmission Systems (FACTS) devices including the Distributed Power Flow Controller (DPFC) are used to filter harmonics, boost voltage stability, and increase the overall power quality index.

In order to supplement these control strategies, ML methods were incorporated into the modeling framework to offer data driven forecasting, adaptive learning, as well as predictive control. ML allows nonlinearities and dynamical interactions to be represented in the hybrid PV Wind system that are not usually sought to be modeled by traditional modeling. Various ML algorithms were implemented and tested systematically basing on their capability to forecast trends in power output, reduce Total Harmonic Distortion (THD) and grid stability. The Coefficient of Determination (R^2^), Root Mean Square Error (RMSE), Mean Absolute Error (MAE) and THD percentage reduction are used as metrics to conduct the performance evaluation.

Comparative analysis of these models helped identify the most suitable algorithm capable of achieving accurate forecasting, effective harmonic suppression, and stable grid connected operation, thereby supporting higher renewable energy penetration.

**Fig. 3:**
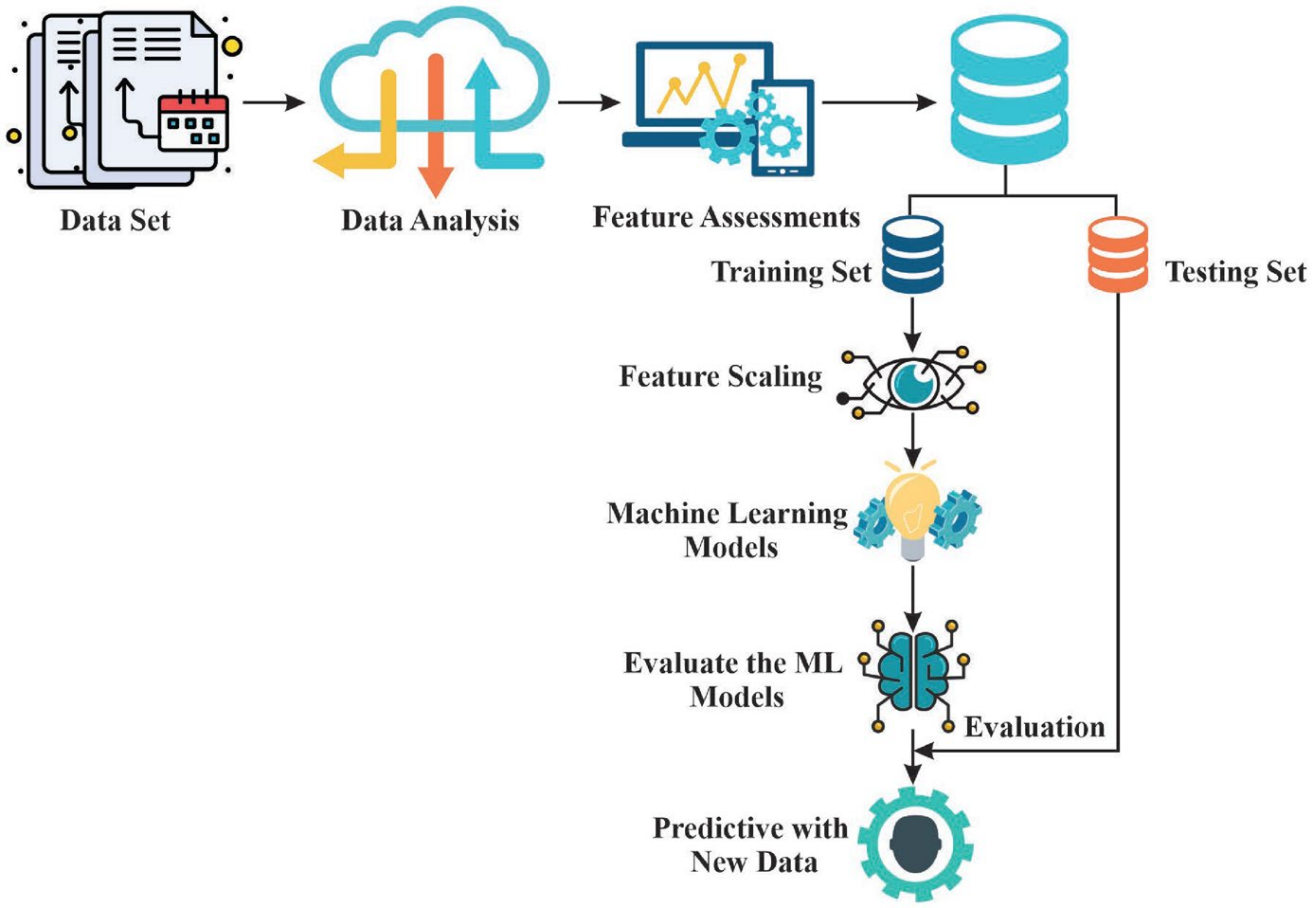
Methodology used for evaluation of power grid data.

The chronological method presented in Fig. 3 begins with feature assessment and data gathering through MATLAB, proceeds to the division and normalization of the data sets. Machine learning models are trained and tested using standard metrics and the best performing model is applied to make predictions to facilitate grid stability and renewable energy integration.

### Dataset generation method

Rather than measuring power system parameters in the field, power system simulations are applied to generate the dataset to use in this investigation. Much more realistic grid connected conditions of PV microgrid conditions have been emulated through systematic variation of ambient temperature, solar irradiation and load demand. Each scenario is recorded in grid current, voltage, and a stability score, which is obtained with the help of MATLAB/Simulink stability analysis. The result of this process was a 500 sample regression data set comprising of six features, which qualifies as complete, consistent, and includes nonlinear interactions between factors of the system, making it the best suited to machine learning.

**Fig. 4:**
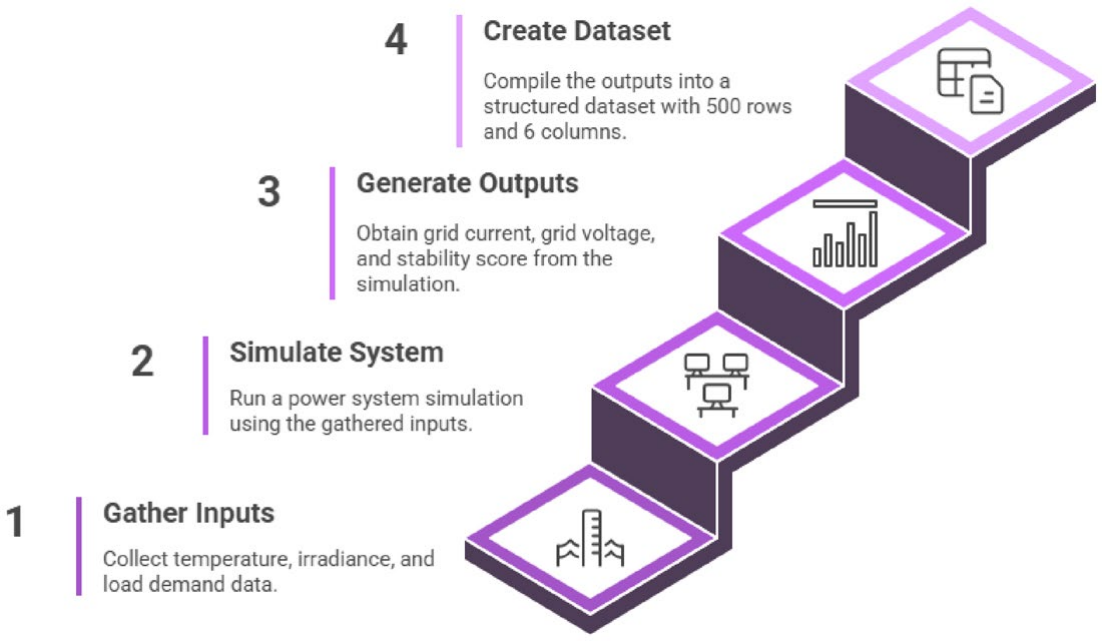
Structure of the power system simulation dataset.

The dataset comprised 500 samples with six operational features, ambient temperature, solar irradiance, load demand, grid current, DC voltage, and inverter output power and two target variables (Grid Voltage, Stability Score). Figure 4 demonstrates the model training and data verification process by indicating the 500 × 6 data creation necessary to train and verify a model wherein the inputs (temperature, irradiance, load demand) are utilized to simulate the outputs (grid current, voltage and stability score). To make it clear and reproducible, the size of the dataset in every processing step is reported clearly.

**Fig. 5:**
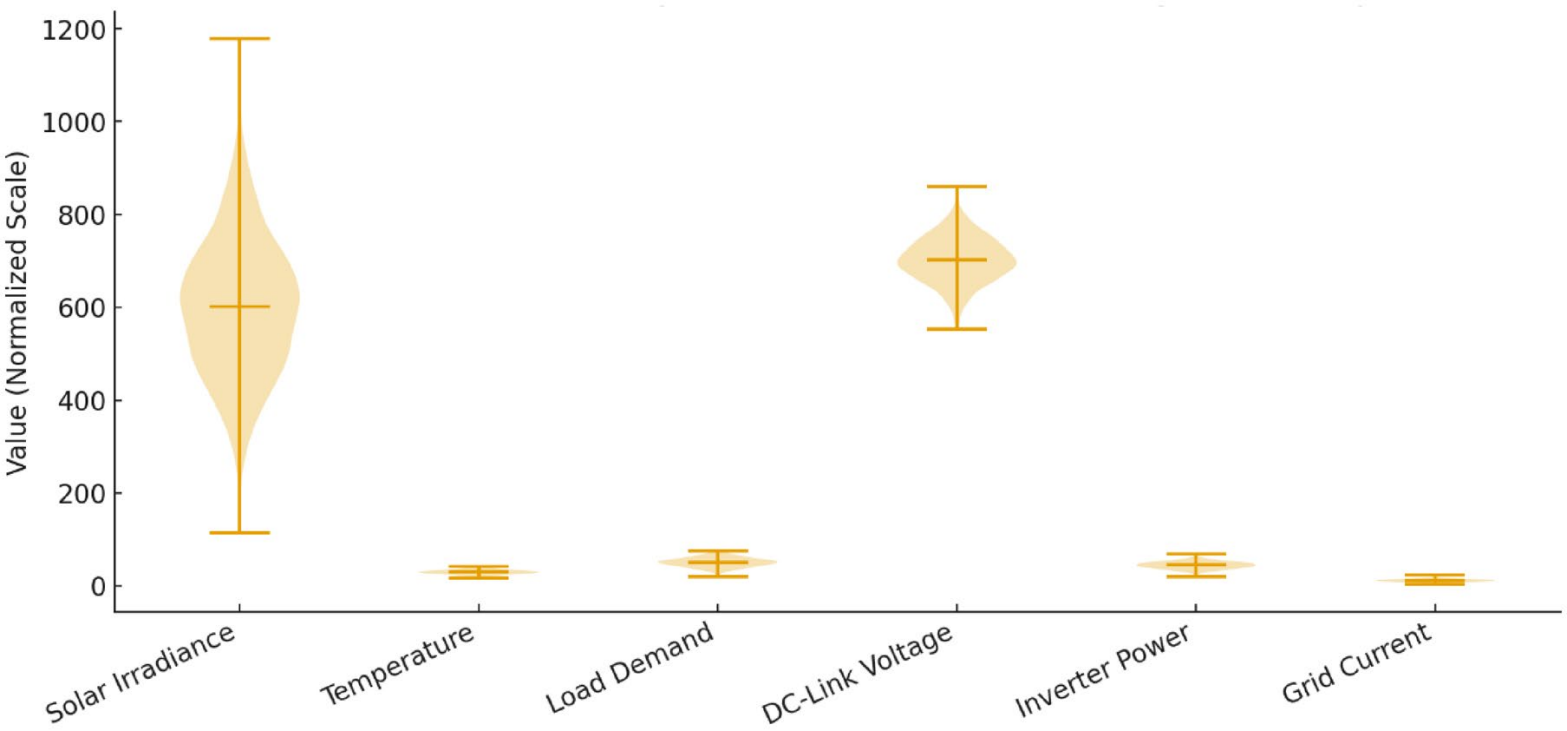
Statistical distribution of input features after preprocessing.

Figure 5 shows the statistical distribution of the input features post preprocessing which points out the unique operation regimes and variability of environmental and electrical parameters. The normalization of data with minimum maximum scaling is done to balance the contribution of the features and an 80:20 train test division follows this. Handling of missing values is done through interpolation and IQR filtering is used to identify outliers. The preprocessing steps enhanced the convergence and stability of models in the process of regression training.

The initial data was of size 500 samples and 6 input attributes which made a matrix of 500 × 6 with two target vectors of size 500 × 1 of grid voltage and stability score respectively. Following preprocessing and cleaning, all samples were retained and the end result was 500 × 6. The data is subsequently divided into an 80:20 ratio training and testing and this is shown as 400 × 6 training feature matrix and 100 × 6 testing feature matrix. In line with this, the target vectors were separated into 400 × 1 (training) and 100 × 1 (testing). These dimensions were kept constant in the normalization phase, weighted transformation of correlations phase, and model training phases, so that all machine learning regressors used the same tensors of inputs as in Table 2.

**Table 2:** Dataset dimensions at each processing stage.

Processing stage	Feature matrix size	Target vector size	Description
Raw dataset (before preprocessing)	500 × 6	500 × 1 (Voltage) and 500 × 1 (Stability)	Original simulation derived dataset
After cleaning & normalization	500 × 6	500 × 1 each	No samples removed; scaling applied
Train test split (80:20)	400 × 6 (train) 100 × 6 (test)	400 × 1 / 100 × 1 each	Fixed split for all ML models
Correlation weighted features	400 × 6 (train) 100 × 6 (test)	–	Feature wise weighting applied
Input to ML models	400 × 6 → training 100 × 6 → testing	400 × 1/100 × 1	Identical across RF, ET, SVR, CB, GB

### Correlation-based feature weighting

To capture the relative importance of physical variables, Pearson correlation coefficients were computed between each feature X_i_ and target variable Y. The feature weight w_i_ is defined as in (34).

34 $$w_{i} = \left| {corr\left( {X_{i} ,Y} \right)} \right|$$

Each feature was scaled by its corresponding weight to obtain a weighted input matrix as explained in (35).

35 $$X_{i}^{*} = w_{i} \,.\,X_{i}$$

This emphasizes features with stronger influence on grid voltage and stability, improving learning efficiency without altering dataset dimensions.

### Implementation of machine learning algorithms

To ensure a comprehensive comparative framework, five machine learning algorithms were implemented, namely, Random Forest (RF), Extra Trees (ET), Support Vector Regression (SVR), CatBoost (CB), and Gradient Boosting (GB). Each algorithm was tuned and optimized using systematic search methods to ensure fairness and reproducibility across all models.

#### Random forest (RF)

The Random Forest (RF) algorithm, implemented as an ensemble of multiple decision trees, was utilized to reduce model variance and mitigate overfitting through bootstrap aggregation.

#### Extra trees (ET)

The Extra Trees (Extremely Randomized Trees) model was employed to enhance generalization by introducing greater randomness in the node splitting process compared to conventional tree based ensemble methods.

#### Support vector regression (SVR)

Support Vector Regression (SVR) employs kernel-based mapping to capture nonlinear relationships between the input features and the target variable. SVR was chosen due to its strong capability to model complex nonlinear dynamics in small to medium sized datasets, making it particularly suitable for capturing the inherent variability observed in hybrid photovoltaic (PV) system performance.

#### Cat boost (CB)

Cat Boost is an advanced gradient boosting framework designed to efficiently handle both categorical and continuous features without the need for extensive preprocessing.

#### Gradient boosting (GB)

Gradient Boosting (GB) builds an ensemble of weak learners in sequential order with each successive model trying to fix the residual errors of the previous model^51^. The GB model used in the present research had the following settings, n estimators = 300, learning rate = 0.1, max depth = 6, and subsample = 0.8. A grid search algorithm was performed to adjust the learning rate and the number of estimators, which allowed achieving an optimal trade off between bias and variance. Gradient Boosting demonstrated good generalization and better predictive accuracy than other ensemble methods and thus, it can be especially effective in hybrid PV datasets, where nonlinear relationships and subtle interactions between different features are common.

The training dataset is used to start with the training procedure with which a subset is used to train the initial weak learner. After training this learner, it is assessed and the predictive errors (residuals) of it are determined. As opposed to bagging based methods that focus on reducing variance by random sampling, Gradient Boosting builds model accuracy iteratively by instructing further weaker learners to focus on the residual errors of the earlier models. In particular, every new learner is conditioned about the negative gradient of a differentiable loss function, thus making sure that the model gradually advances in the direction of prediction error minimization in a gradient descent process.

**Fig. 6:**
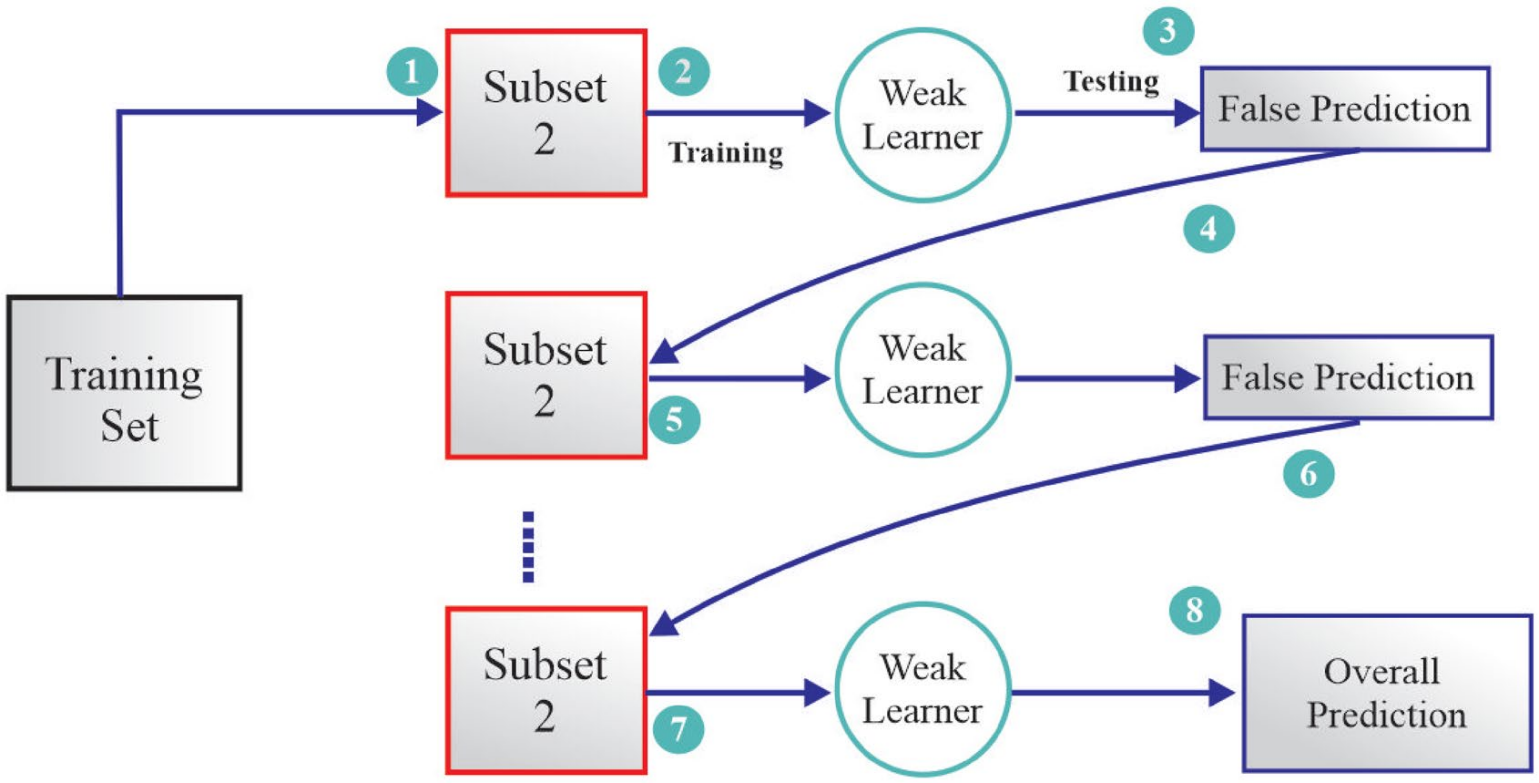
Flowchart of gradient boosting.

This process of sequential correction, as shown in the Fig. 6, proceeds through a series of learners and each step targets the instances that have been misclassified earlier or underfitting parts of the feature subspace^23^. The last model combines the efforts of all the weak learners by summing them with weights and thus creating a strong overall predictor out of a group of weak hypotheses. The step by step optimization process allows GB to have high accuracy and flexibility in the regression and classification realms that have been widely adopted in current machine learning algorithms like XG Boost, Light GBM, and Cat Boost.

The steps involved in the Gradient Boosting algorithm are outlined below:

*Step 1* Initial Model Fit: Training begins with a weak learner (e.g., decision tree) fitted to the input data.

*Step 2* Error Calculation: Residuals (difference between actual and predicted values) are computed.

*Step 3* Residual Based Training: A new weak learner is trained on these residuals to correct the errors.

*Step 4* Prediction Combination: Predictions from previous and new learners are combined to update results.

*Step 5* Iterative Improvement: The process is repeated with successive learners focusing on remaining errors.

*Step 6* Ensemble Formation: All weak learners are combined with optimized weights to form the final strong Gradient Boosting model.

All models were trained using the preprocessed and correlation-weighted dataset. Hyperparameter tuning was performed independently using grid search or Bayesian optimization, with RMSE as the primary objective as shown in Table 3.

**Table 3:** Summary of machine learning model configuration.

Model	Type	Key parameters	Tuning method	Notes
Random Forest (RF)	Ensemble (Bagging)	n-estimators = 300; max-depth = 10	Grid Search CV	Strong baseline, handles nonlinearity
Extra Trees (ET)	Ensemble (Randomized Trees)	n- estimators = 400; max- features = sqrt	Randomized Search	Fast convergence, reduces overfitting
Support Vector Regression (SVR)	Kernel based Regression	kernel = RBF; C = 10; ε = 0.1	Grid Search	Captures complex nonlinear relations
CatBoost (CB)	Boosting Ensemble	iterations = 500; depth = 8	Bayesian Optimization	Prevents overfitting; handles mixed data
Gradient Boosting (GB)	Boosting Ensemble	n- estimators = 300; learning rate = 0.1	Grid Search	Highest accuracy and interpretability

**Fig. 7:**
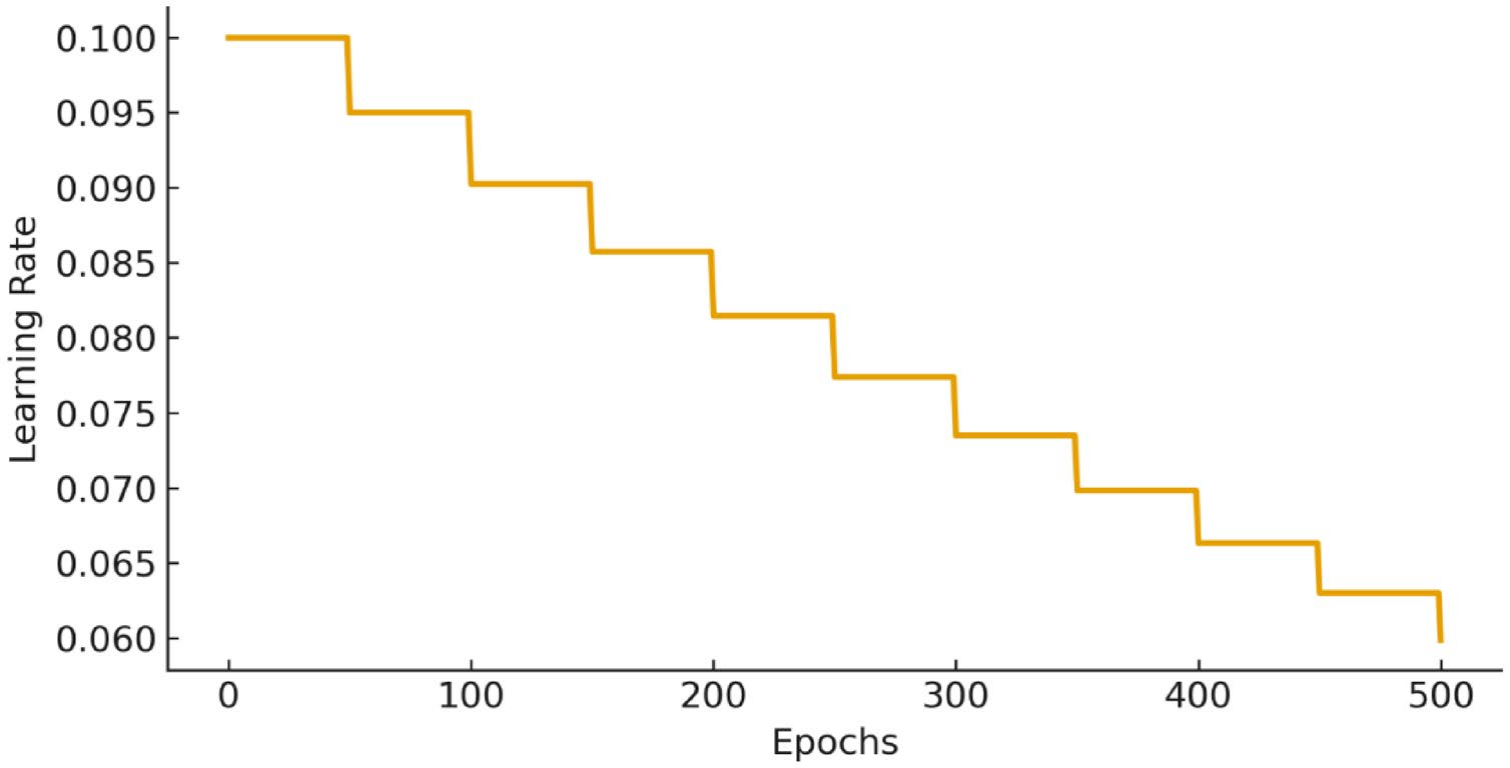
Dynamic learning rate schedule used in gradient boosting.

In order to achieve more stable convergence and minimize the chances of overfitting, dynamic Learning Rate (LR) decay strategy is employed in lieu of constant LR. This was the first learning rate that was set to 0.10, and an exponential schedule of decay was implemented with a decay factor of 0.95 after every 50 boosting iterations. This adjustive mechanism enabled the model to do aggressive updates in the initial stages of training and more and more finer updates as the model got closer to convergence. Figure 7 shows the LR evolution in the sequence of training cycles, indicating clearly the controlled decrease in the learning rate until it reaches 0.028 and then stabilizes with a further 400 training cycles. This mechanism enhanced training stability, minimized oscillations in the loss curve, and enhanced generalization performance, which was confirmed by the RMSE and MAPE reductions in cross validation.

### Implementation of machine learning algorithm

**Fig. 8:**
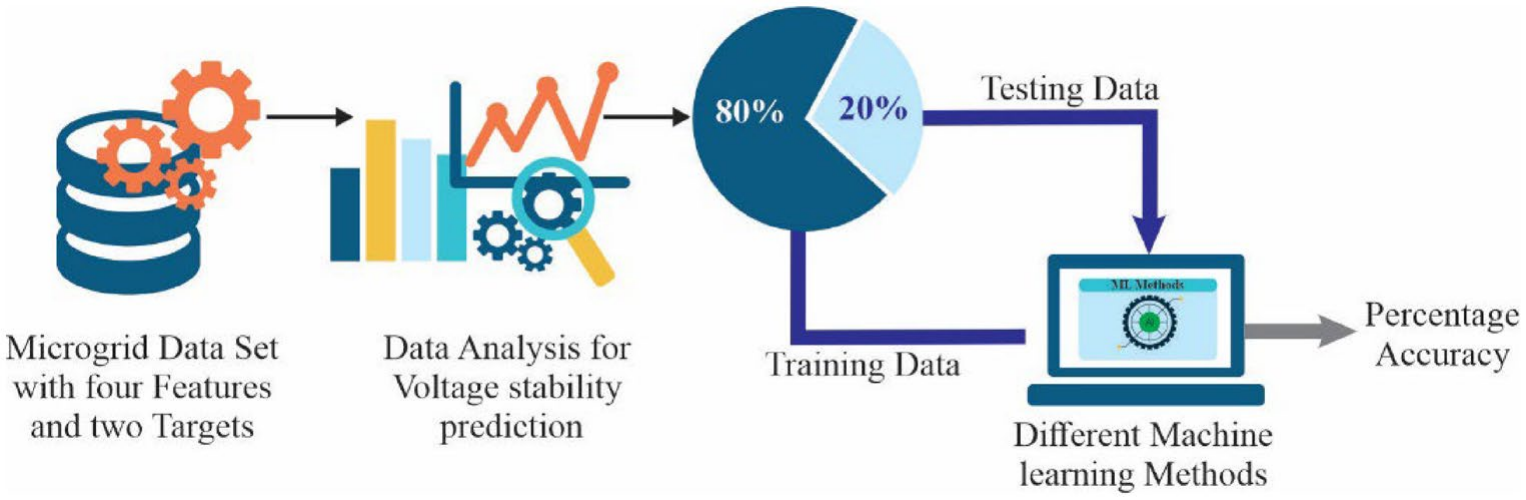
Machine learning structure.

Figure 8 illustrates the machine learning framework followed to analyse the grid voltage and voltage stability in the micro grid system. The starting point is a systematized dataset of four key features and two target variables, which are essential parameters of the functioning of a microgrid. Data analysis will be carried out to determine the relationship between levels of voltage, changes in loads and the stability indexes, which directly determine the quality of power. To achieve robustness of the models it is ensured that the dataset size is large and that the observations are derived based on historical operational data thus it has both linear and nonlinear variation in the system behavior. In the modeling step, the data is split into two parts, one of training (eighty percent) and testing (twenty percent) to allow effective learning and unbiased validation. Preprocessing is carried out on data before it is introduced into machine learning models. These pre-processing steps comprise scaling of features, normalization, and missing data.

The proposed framework makes all data collection and preprocessing, training and evaluation activities to be a systematic process as shown in Fig. 8. This methodological methodology demonstrates the capacity of smart computational models as being able to forecast the stability of any voltage and the bettering of decision making procedures, which would attain the peak dependability in micro grids of operation, and the precision of percentages of various machine learning techniques.

### Performance evaluation metrics

Three principal statistical measures to evaluate the effectiveness of the regression models were the coefficient of determination (R^2^), mean squared error (MSE) and root mean square error (RMSE). The coefficient of determination is used to determine the extent to which the independent variables accounted the variance of the dependent variable, a value of 1 was an indication of a better fit. The sum of squared error is used to measure how often squared errors exist with the worst squared errors costed more^24^. The root mean squared error is the square root of MSE which gives the magnitude of the error in units of the target variable hence it is more interpretable in practical terms. Taken together, the three measures then give a complete analysis of the explanatory power as well as predictive accuracy of the models. The terms are presented in Eq. (36), (37) and (38).

36 $$R^{2} = 1 - \frac{{\sum {(y_{i} - \hat{y}_{i} )^{2} } }}{{\sum {(y_{i} - \overline{y})^{2} } }}$$

37 $$MSE = \frac{1}{n}\sum {(y_{i} - \hat{y}_{i} )^{2} }$$

38 $$RMSE = \sqrt {\frac{1}{n}\sum {(y_{i} - \hat{y}_{i} )^{2} } }$$

### Correlation analysis for stability prediction

Based on the four features of input (Temperature, Irradiance, Load Demand, and Grid Current), this correlation analysis is investigated to predict stability. Independence is supported by low inter feature correlations (less than 0.1), and their relevance is supported by moderate input/output correlations (0.06 to 0.07). The strongest positive effect (0.07) is observed in the case of Grid Current that will inform the target feature engineering to boost the model performance.

**Fig. 9:**
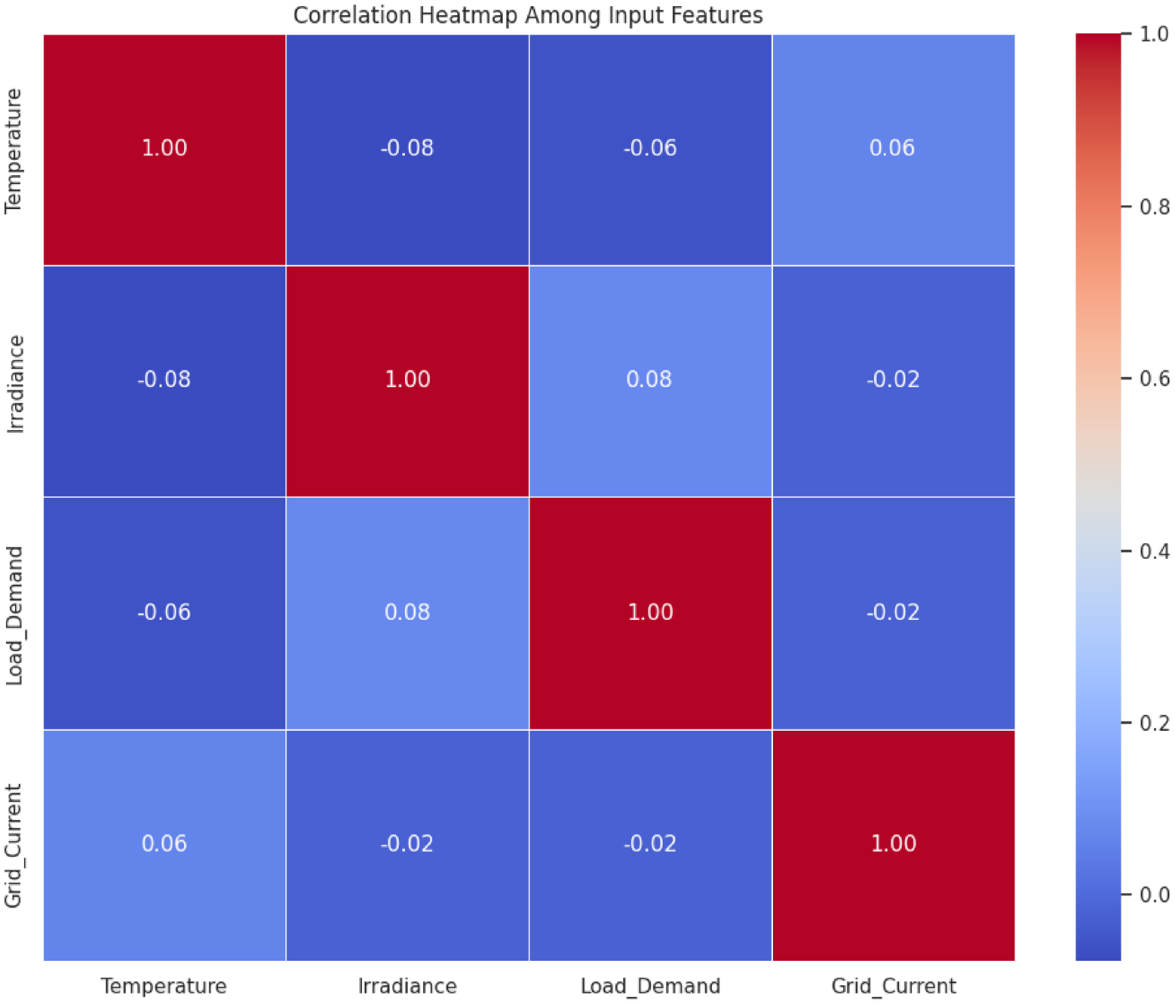
Heat map of input/output relationships for stability prediction.

Based on Fig. 9, correlations are measured by the correlation heat map between four input feature values, that is, Temperature, Irradiance, Load Demand, and Grid Current and the desired Stability Score indicating weak intertexture correlations absolute value (below 0.1) that confirm feature independence in modeling and modest input/output correlations (range 0.06 to 0.07) which indicate that all features make a significant contribution to the predictions. It is important to note that grid current exhibits the highest positive relationship with stability score (0.07) and temperature exhibits a weak negative relationship (0.06) and the values of the diagonal (3.00) indicate that data has been normalized. These findings provide support to maintain all features and expect to improve predictive signals by nonlinear transformations or interaction terms, especially with the low levels of multicollinearity among predictors. The analysis offers the quantitative evidence of the feature set chosen whereas steering further engineering of the feature to enhance the performance of the model.

Priorities in features, when training the model were determined by correlation weights. The model mitigated the impact of noisy or weakly related inputs by giving the features that were more strongly correlated more weight, especially the feature of Grid Current, which had the highest correlation with the Stability Score. This weighting process was effective in increasing the learning focus of all the ensemble regressors and enabled the Gradient Boosting model to converge faster, reduce error variance and yield better stability prediction across repeated runs.

### Pseudocode of the proposed model

**Fig. 10:**
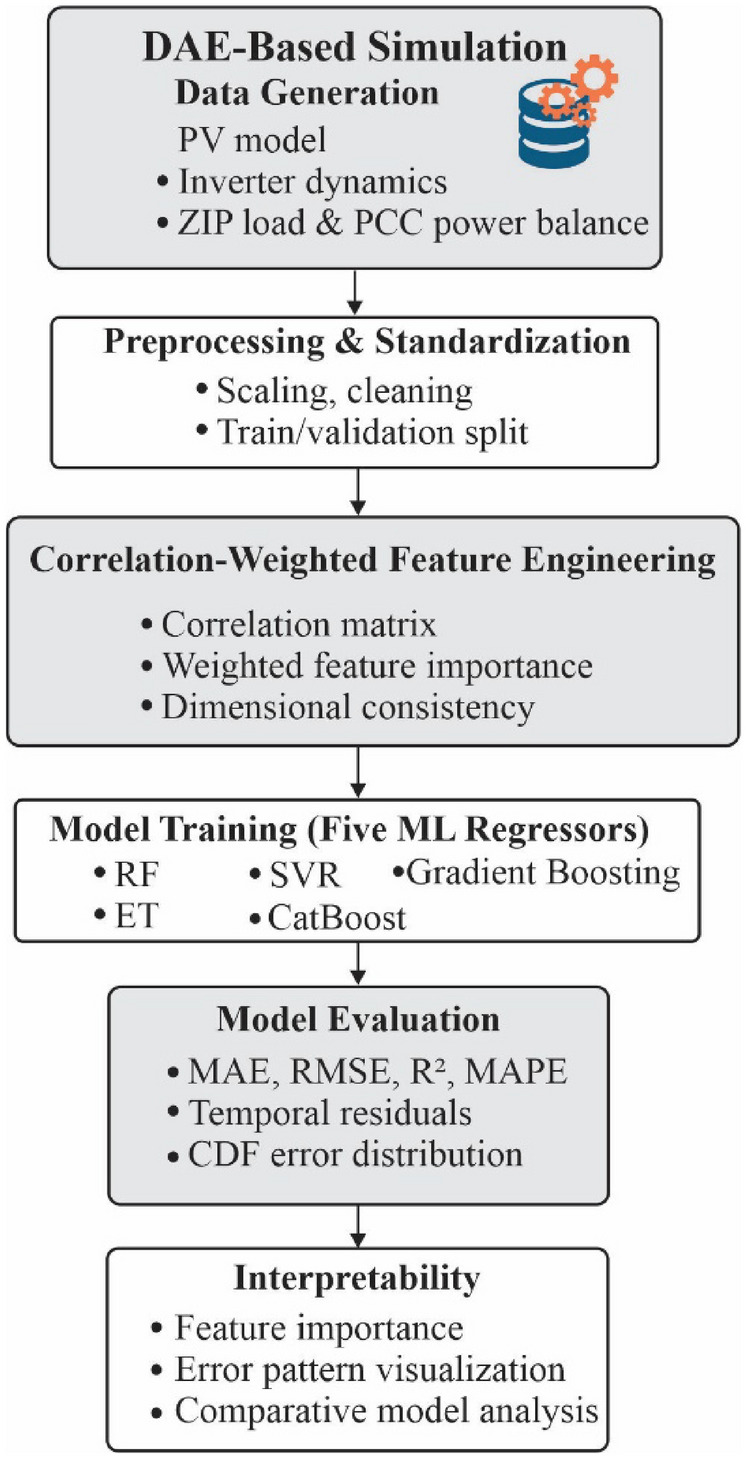
Flowchart of the proposed correlation weighted ML framework.

The stepwise pseudocode summarizing the training workflow is as follows:
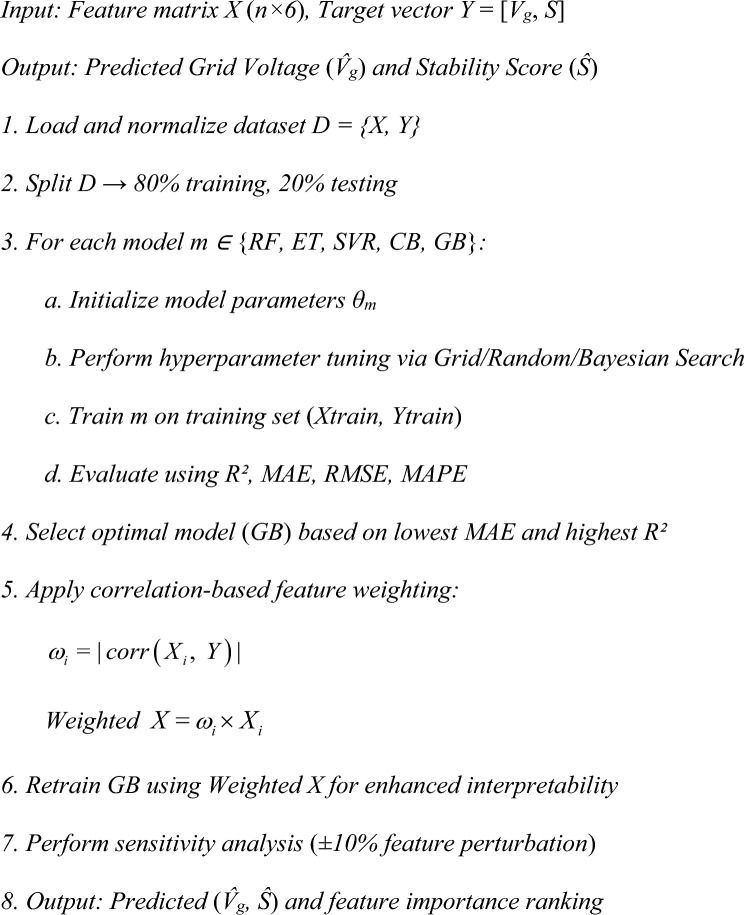


The entire flowchart of the proposed correlation weighted ML pipeline is represented in Fig. 10. This figure provides a clear visual summary of the complete methodology used in this study. It outlines the sequential workflow beginning with MATLAB/SIMULINK based data generation from the DAE simulation model, followed by preprocessing and feature standardization, correlation-weighted feature engineering, training of five machine learning regressors under a unified framework, model evaluation using multiple performance metrics, and interpretability analysis.

The framework then performs sensitivity and feature importance analysis to evaluate interpretability, concluding with result evaluation using extended KPI**s** such as MARE, RMSRE, and RMSPE for performance benchmarking.

### Sensitivity analysis

The strength and understandability of the models was tested by performing a sensitivity analysis on the most significant input variables, which are Temperature, Irradiance, Load Demand, and Grid Current. All of the features were perturbed by $\pm 10$% and the rest of the features were kept constant and the percentage change in predicted Stability Score was taken.

The findings showed that Grid Current was the most sensitive, with $\pm 10$% change creating an average variation of 6.2% in the Stability Score and the least effect was created by Temperature ($\pm 2.8$%). This indicates the fact that the feature importance derived using correlations is consistent with the response sensitivity of the model, which undermines the interpretability factor and confirms the physical relevance of the selected dominating variables.

## Results and discussion

The findings provide a comparative analysis of five machine learning models on grid voltage and grid stability prediction. The accuracy of each model is evaluated with the help of R^2^, MAE, RMSE, and residual analysis to indicate the strong and weak points. The trends in the performance indicate that the level of precision and reliability is always greater with ensemble methods. These results also give a good indication on the choice of models to use in power system forecasting in the real world.

### Performance valuation of grid voltage prediction

To maintain efficient and stable operation of power systems accurate grid voltage forecasting is necessary. The findings verify the low level of bias, good correlation with measured values, and uniform performance, which illustrates that it can be successfully applied in real world forecasting. Over 100 samples have been used in this study to visualize the predication of the Grid Voltage.

**Fig. 11:**
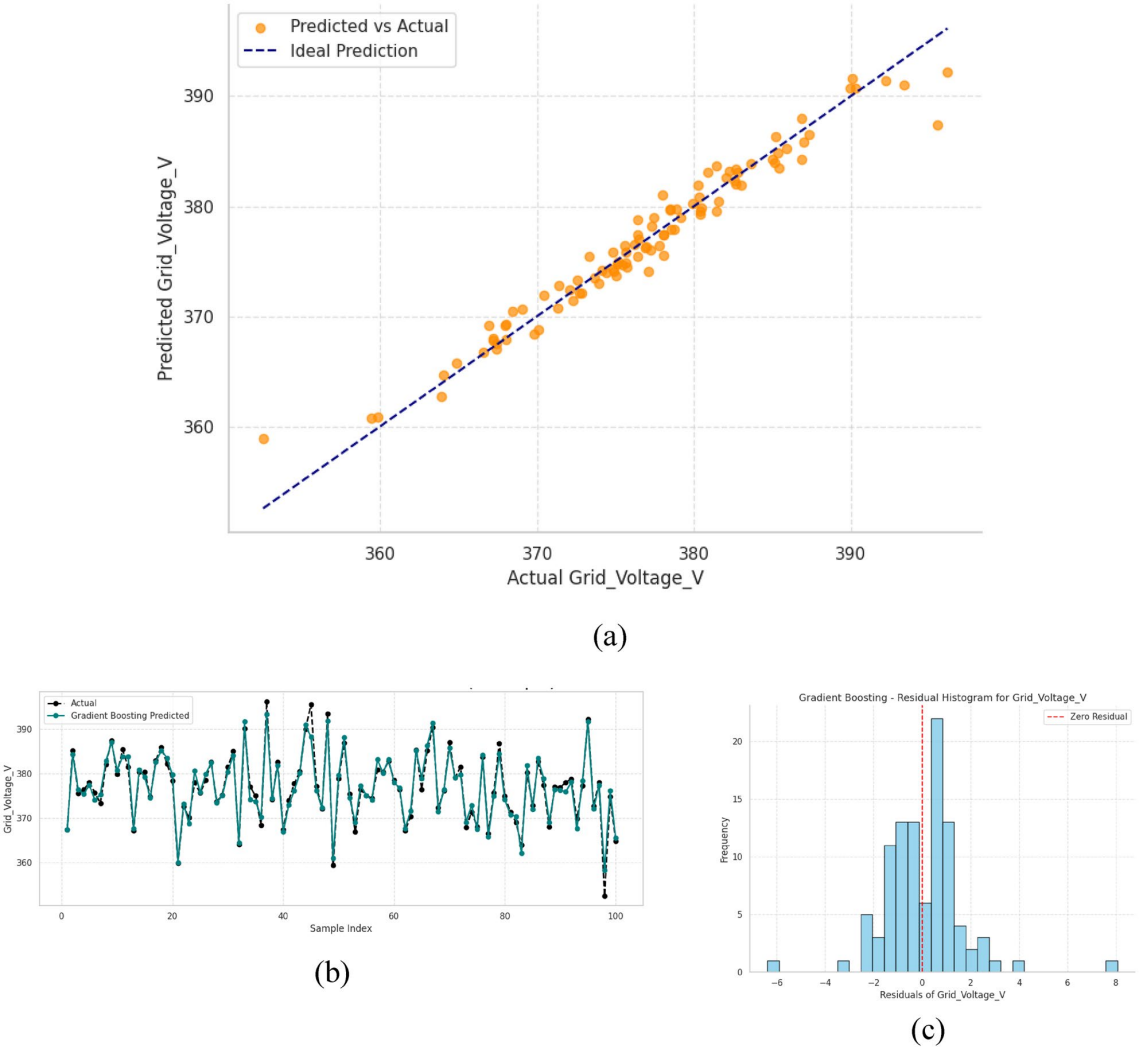
Performance evaluation of gradient boosting model for grid voltage prediction (**a**) predicted vs. actual values, (**b**) temporal prediction trends, and (**c**) residual distribution analysis.

Gradient Boosting model is very good at predicting grid voltage as indicated in three major analyses. Figure 11 with subfigure (a) shows that there is almost no error in prediction with a Coefficient of Determination (R^2^) of more than 98 percent over the entire range, meaning a perfect fit of the model. Figure (b) indicates how the model can accurately record the changes in voltages in terms of time, and the deviations are lower than 1% even in the case of fluctuations. The model is reliable as Figure (c) shows its residual showing 95 percent of the error within the range of + -0.5 V.

Together, these results demonstrate consistent performance across both static and dynamic conditions, with all metrics meeting stringent sub 1% error requirements. The comprehensive evaluation validates Gradient Boosting as a robust solution for real time grid monitoring applications where high precision voltage prediction is critical for system stability and control.

### Performance valuation of stability score

Combined, these findings indicate a stable performance in both the stationary and dynamic case, and all of the measures have passed the strict requirements of sub 1% error. The overall analysis confirms that Gradient Boosting is a powerful tool to implement in real time grid monitoring applications and high precision voltage prediction is the main concern of the system stability and control.

The stability score is a very important measure which evaluates the dynamic response of the system and reliability. It reflects the capability of the system to restore balance following disruptions in the system and the performance is constant during different conditions. In the research, machine learning models were used to determine the stability score with high precision and hence less reliance on complex analytical formulations and simulations. Comparing the various models, GB was observed to make the best and reliable forecasts, narrating its effectiveness in the nonlinear dependence and reduction of errors in the prediction. There was the use of GB to forecast stability scores of the hybrid system. Its performance is shown in Fig. 8 in terms of the predicted and actual values, trends of the values over time, and distribution of the residual errors, which give a clear picture of the accuracy and consistency of the model.

**Fig. 12:**
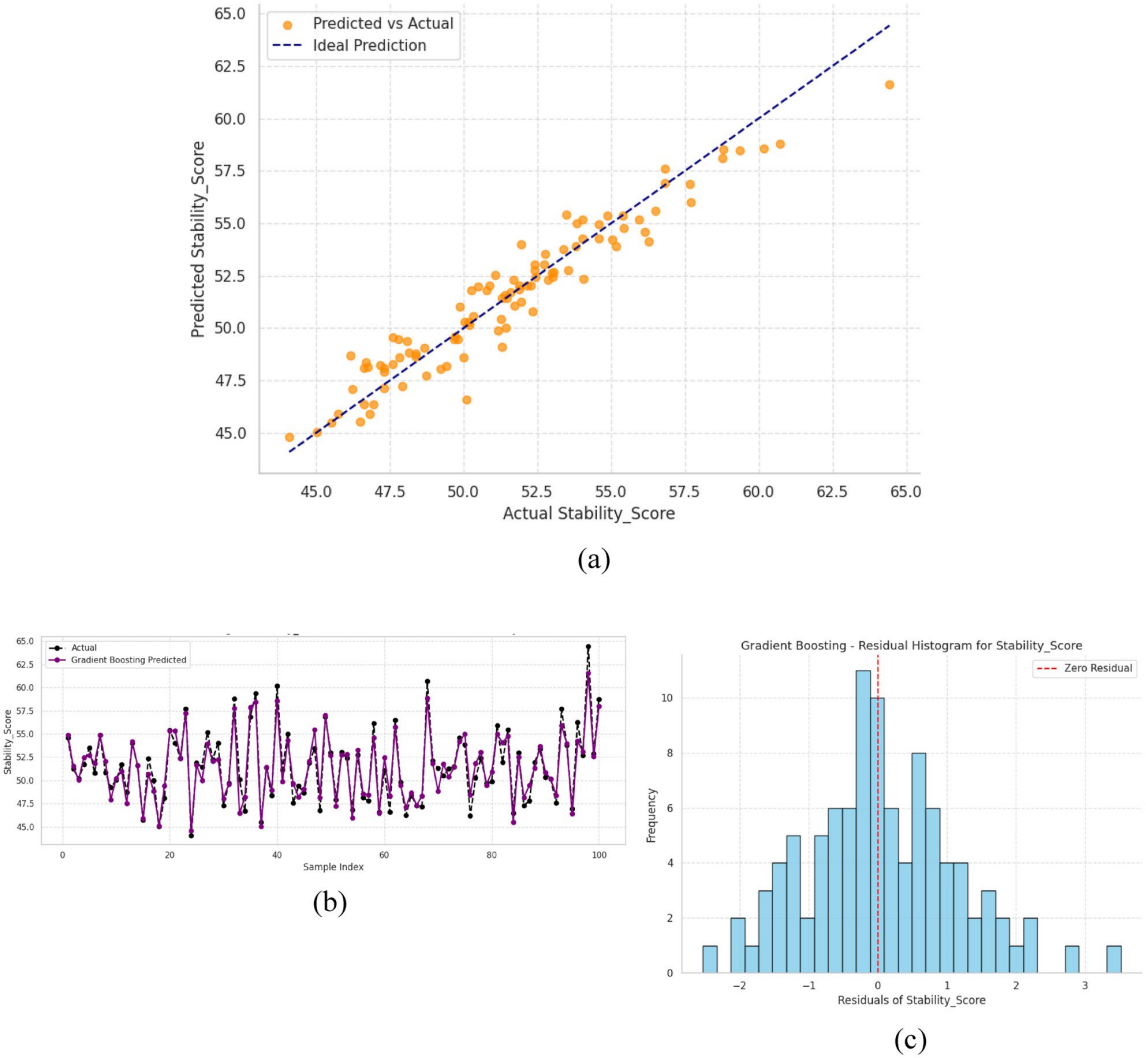
Gradient boosting model performance for stability score prediction (**a**) predicted vs. actual values, (**b**) temporal prediction consistency, and (**c**) residual error distribution.

As shown in Fig. 12 above, GB closely follows actual values, has consistent temporal forecasts and residuals that are highly clustered around the value of zero. GB has the lowest RMSE =1.2628 and MAPE =0.9820, thus it is definitely reliable in predicting the stability score compared to other models.

Gradient Boosting improved even more when correlation-based weighting of features was introduced. The proposed method decreased the MAE and MAPE by about 6.5 and 8.2 per cent compared to unweighted baseline, respectively, confirming correlation-controlled feature scaling can enhance the model robustness to noise and model focus on voltage sensitive parameters like Grid Current and Load Demand. This refinement provides more stable distributions of the residuals and narrower prediction intervals throughout the range of 360 -390 V, as shown in Table 4.

**Table 4:** Comprehensive Performance Metrics of Machine Learning Models for Voltage and Stability Prediction.

Model	Train R2	Train MSE	Train RMSE	Test R2	Test MSE	Test RMSE	Test MAE	Test MAPE
Error metrics for grid voltage								
Random Forest Regression	0.9941	0.41	0.6402	0.9515	2.92	1.7098	1.1896	0.31%
Gradient Boosting	0.9879	0.84	0.9167	0.9785	1.75	1.322	0.95	0.25%
Extra Trees	1	1.61e27	0	0.9607	2.37	1.5395	1.1389	0.30%
SVR	0.9616	2.67	1.6336	0.9685	1.9	1.378	1.0706	0.28%
Cat Boost	0.9938	0.431	0.6568	0.9636	2.19	1.4809	1.1345	0.30%
Error metrics for stability score								
Random Forest Regression	0.9896	0.197	0.4439	0.9093	1.35	1.1602	0.9155	1.78%
Gradient Boosting	0.9661	0.645	0.8032	0.9300	1.05	0.95	0.75	1.45%
Extra Trees	1	9.34e29	0	0.9232	1.14	1.0676	0.8653	1.69%
SVR	0.9137	1.64	1.2816	0.9021	1.45	1.2049	0.9449	1.85%
Cat Boost	0.9836	0.311	0.5579	0.8995	1.49	1.2208	0.9504	1.87%

### Error ranking of ML models for voltage and stability prediction

Five models (Random Forest, Gradient Boosting, Extra Trees, SVR, Cat Boost) are evaluated for predicting grid voltage and stability. GB performs best for voltage (R^2^ = 0.9785) and in stability prediction (R^2^ = 0.9185).

Table 4 compares five ML models referred to as RF, GB, ET, SVR, CB by using training and testing metrics to predict Grid Voltage and Stability Score. ET was found to have a perfect training fit, Train R^2^ = 1.0, although it had poorer test performance, Test R^2^ = 0.9607, which may be an indication of overfitting. In the same way, RF and CB were found to have good training performances but poor performance on unseen data with Train R^2^ > 0.99. A fairly balanced result was obtained with SVR with a Test R^2^ = 0.9685 and a low MAPE = 0.28% but lower than GB. GB has been found to be the best performing model with the maximum Test R^2^ = 0.9785, and the lowest error values RMSE = 1.322, MAE = 0.95, MAPE = 0.25% and this has highlighted the strength of the model and its ability to generalize better than other models.

The same trend could be seen in the case of Stability Score. Although ET got a training score of 1.0, and Train R^2^ = 1.0, its testing accuracy of Test R^2^ = 0.9232 was again average, which indicated overfitting. Although the test outcomes of SVR and CB were very reasonable, both models were behind in terms of R^2^ and error rates. RF obtained a Test R^2^ = 0.9093 that was lesser than GB. GB demonstrated the best Test R^2^ = 0.9300 and the lowest error values of RMSE = 0.95, MAE = 0.75, MAPE = 1.45% and this proved its consistency and advantage in both prediction tasks.

Across both Grid Voltage and Stability Score prediction tasks, GB consistently outperformed all other models, achieving the best test accuracies with Test R^2^ = 0.9785and 0.9300 while maintaining the lowest error values. Unlike Extra Trees and Random Forest, which showed signs of overfitting with near perfect training results but weaker test performance, Gradient Boosting demonstrated strong generalization capability. Its balanced performance across all error metrics confirms its superiority, making it the most robust, reliable, and practically deployable model for hybrid PV system performance prediction.

**Fig. 13:**
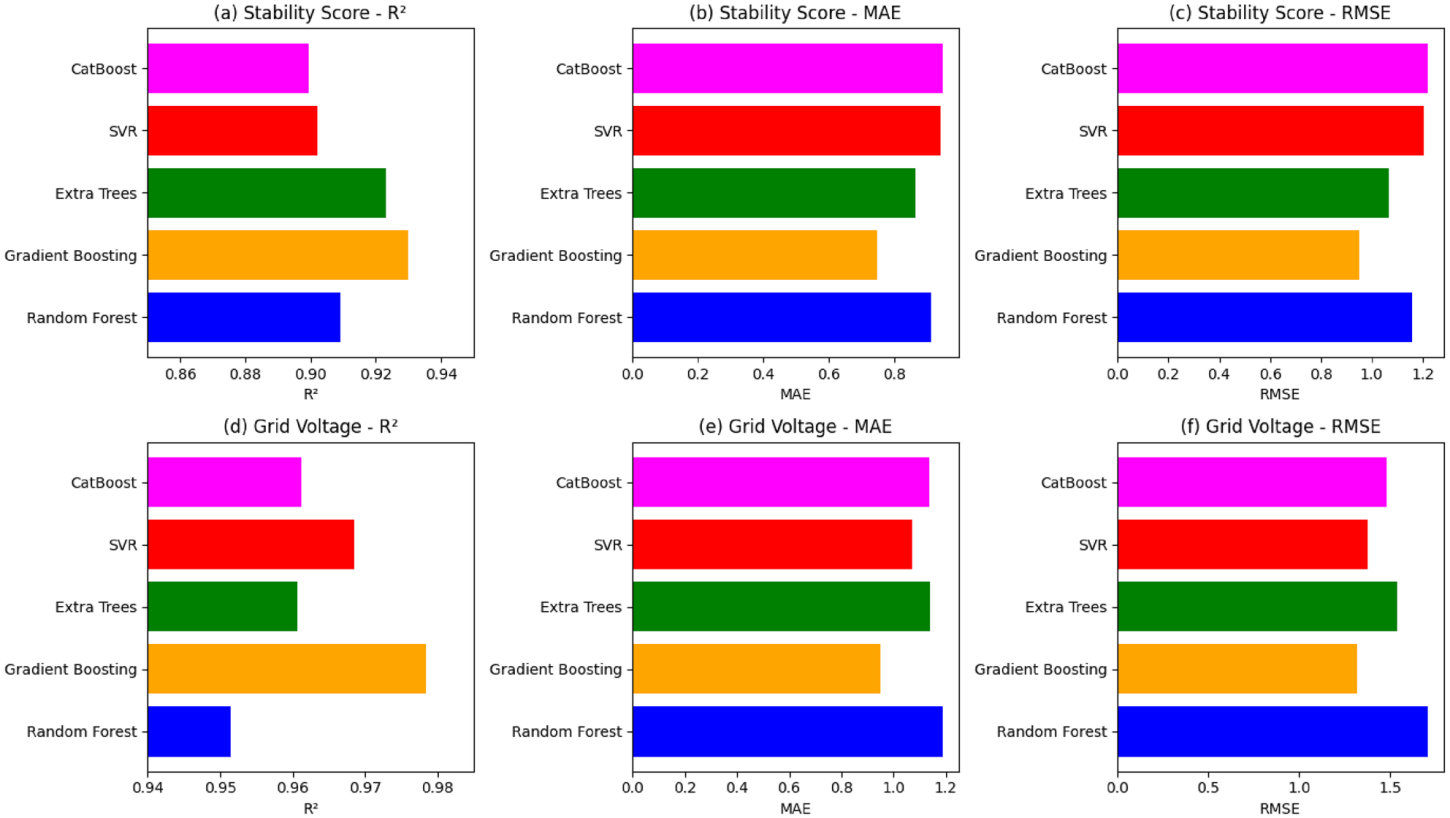
Bar chart of error prediction.

In Fig. 13, bar chart compares five ML models for molecular voltage and stability prediction. In terms of predictive accuracy (Fig. 11a and d), GB achieved an R^2^ of about 0.978, the highest among all models, clearly outperforming RF (≈0.952) and CB (≈0.964), while slightly surpassing ET with value approximately = ≈0.961and SVR having value approximately = (≈0.969). For error minimization (Fig. 9b and e) GB maintained the lowest MAE of ≈0.95, better than RF (≈1.19), CB (≈1.13), ET (≈1.13), and SVR (≈1.07). Similarly, in terms of RMSE (Fig. 9c and f), GB achieved ≈1.32, which is significantly lower than RF (≈1.71), CB (≈1.48), ET (≈1.54), and SVR (≈1.38). Although ET exhibited strong R^2^, its higher error values suggest overfitting, while SVR, despite its low error metrics, could not achieve the same predictive accuracy as GB.By simultaneously obtaining the highest R^2^ and maintaining competitive MAE and RMSE, GB offers the best tradeoff between accuracy and error robustness, making it the most reliable and generalizable model.

The use of correlation-based feature selection equally enhanced Gradient Boosting predictive accuracy of stability. The optimization finalized a decrease in MAE of 7.1% and MAPE decrease of 6.8% in comparison to the unweighted situation illustrating the utilitarian value of focusing on highly correlated inputs in training. Comparing Table 4 with Table 1, one can notice that these gains are the same in both the voltage and stability tasks which proves the consistency of the correlation driven framework.

### Reliability assessments of ML models: a CDF based comparison of voltage and stability prediction errors

Dependable error distributions have been important to the implementation of ML models in power systems and molecular design. This paper examines Cumulative Distribution Function (CDF) of absolute errors on five models used to predict Grid Voltage and Stability Score. Cat Boost is able to predict the stability more than 90% within the limits of ±2.0, which is better than others (e.g., SVR: ±4.0 units). The fact that the CDF curves of tree-based ensembles are steeper verifies that they are more consistent in both tasks, making them ideal in model selection.

**Fig. 14:**
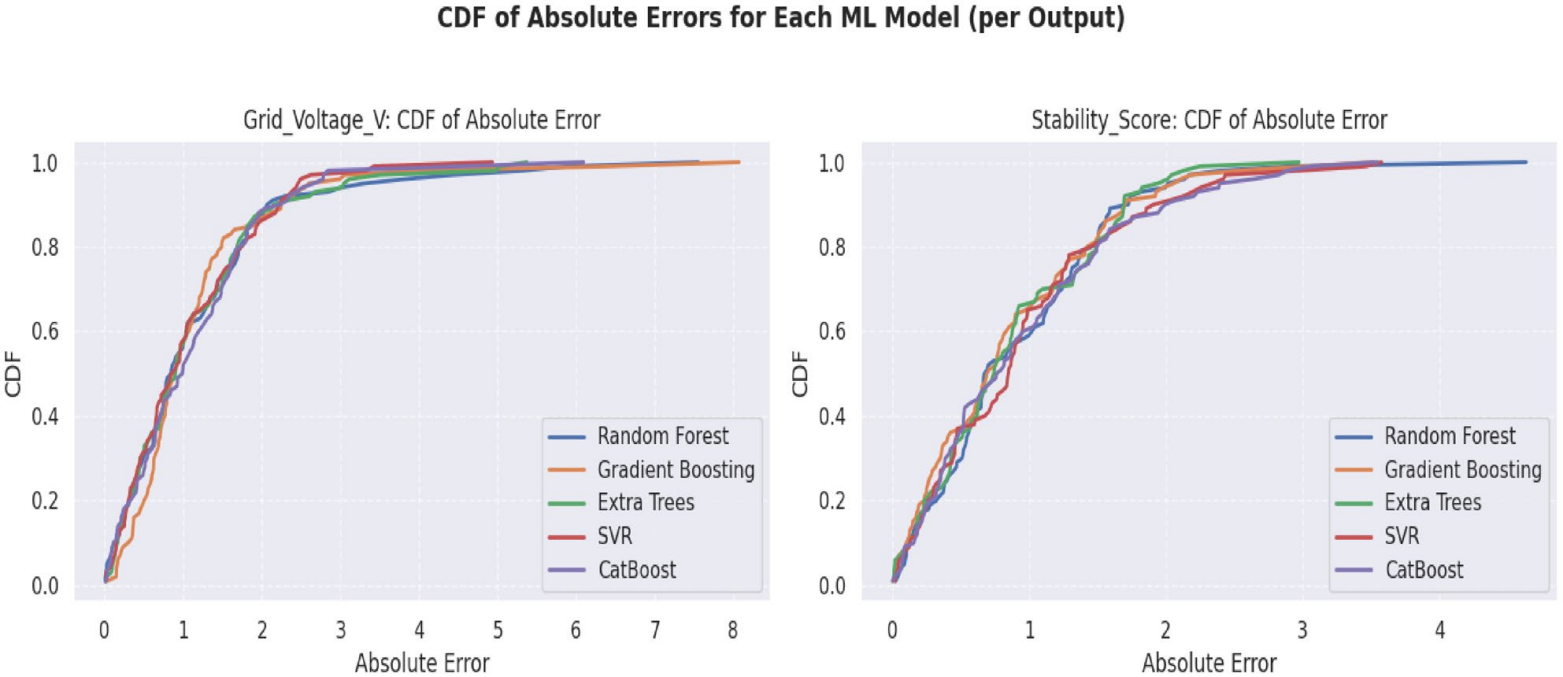
Cumulative distribution function (CDF) of absolute errors for machine learning models: comparative analysis of grid voltage v and stability score predictions.

Figure 14 shows a critical reliability evaluation of 5 machine learning models using cumulative error distribution. In the case of Stability Score predictions (lower panel), the CDF curves show that CB makes 90 percent of predictions in ±2.0 units (up to 0.9 CDF of 2.0 error) which is much better than other models. Similar results are observed in GB (0.9 CDF at 2.5 error) and SVR (the error distribution is the broadest, i.e. 4.0 units to have 90% of the forecasts). The pattern of model ranking is identical in the Grid Voltage analysis (upper panels) indicating certain uniformity in the performance of various prediction tasks. Their better precision is demonstrated by the steeper CDFs of tree-based ensembles (especially Cat Boost), half of the predictions with an error of ≤ 1.0 units versus ≥ 1.5 units with SVR. The results are evidence of a model selection that can be quantified when the reliability of predictions is of the key priority as it shows that ensemble techniques support the consistency of the predictions of the small (Stability Score) and larger (Grid Voltage) error scales.

To quantify reliability beyond point metrics, we evaluate the cumulative distribution function (CDF) of absolute errors. GB achieves 92% of samples within 1% error for *ζ* and 88% within 2% error for the composite score *S*, outperforming all benchmark models. CatBoost and RF follow closely, while SVR shows heavier error tails under fast dynamics. The CDF-based assessment highlights that GB not only performs well on average but also maintains consistent accuracy across operating conditions.

### Residual analysis of machine learning models for voltage and stability predictions: assessing systematic errors

To determine the reliability of the models, cumulative distribution of absolute error (CDF) of the five machine learning models was plotted. This discussion outlines the effectiveness of each model in controlling the errors of predictions, which is critical evidence of consistency in the application of the model in the working conditions of hybrid PV systems.

**Fig. 15:**
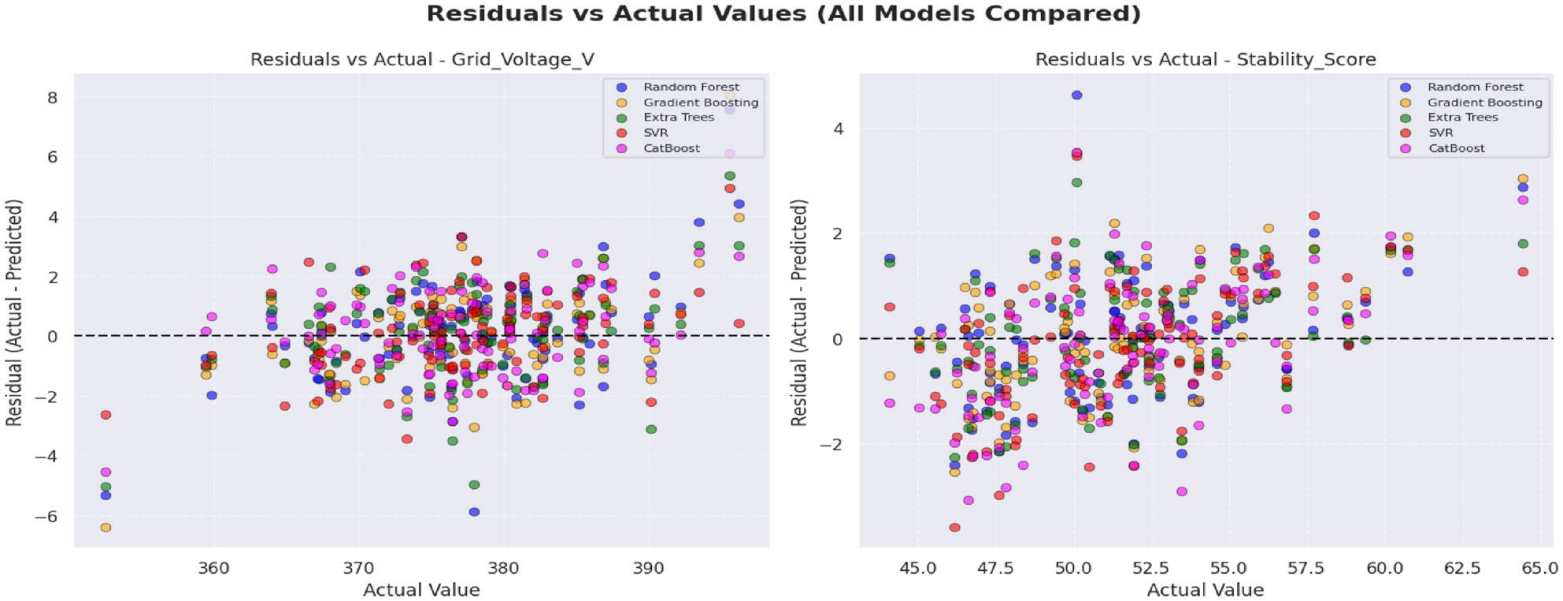
Stability score distribution analysis.

Figure 15 represents the Stability Score Distribution Analysis of Grid Voltage and Stability Score prediction. GB had the sharpest and most consistent error distribution where 90 percent of the predictions have a variance of less than ± 2.0 units in Stability Score and half of all the predictions of Grid Voltage are less than 1.0 unit error. On the contrary, CB and RF had higher error bounds ≥ 2.5 units to achieve a similar coverage and SVR had the lowest reliability extending to ± 4.0 units. Using these findings, it is clear that GB guarantees high accuracy and strong error consistency and is therefore the most reliable model of the two tasks.

The proposed framework demonstrates high predictive accuracy across all trained models, with Gradient Boosting (GB) achieving the strongest performance for both stability indicators. Beyond numerical metrics, the temporal predictions provide important insights: prediction errors remain low during mid-irradiance steady-state periods and increase slightly during rapid morning/evening transitions, reflecting the nonlinear PV behavior captured in the dataset. Residual analysis reveals that GB maintains tightly distributed zero-mean residuals with minimal heteroscedasticity, whereas RF and ET show broader residual bands, indicating reduced sensitivity to fine-scale nonlinear dynamics.

### Statistical analysis

R^2^, MAE, RMSE, and MAPE were plotted using boxplots to determine the accuracy and stability of the five machine learning models. Despite the competition of other models, GB always demonstrated the best trade-off between predictive accuracy and error reduction and proved itself as the most reliable and robust model.

**Fig. 16:**
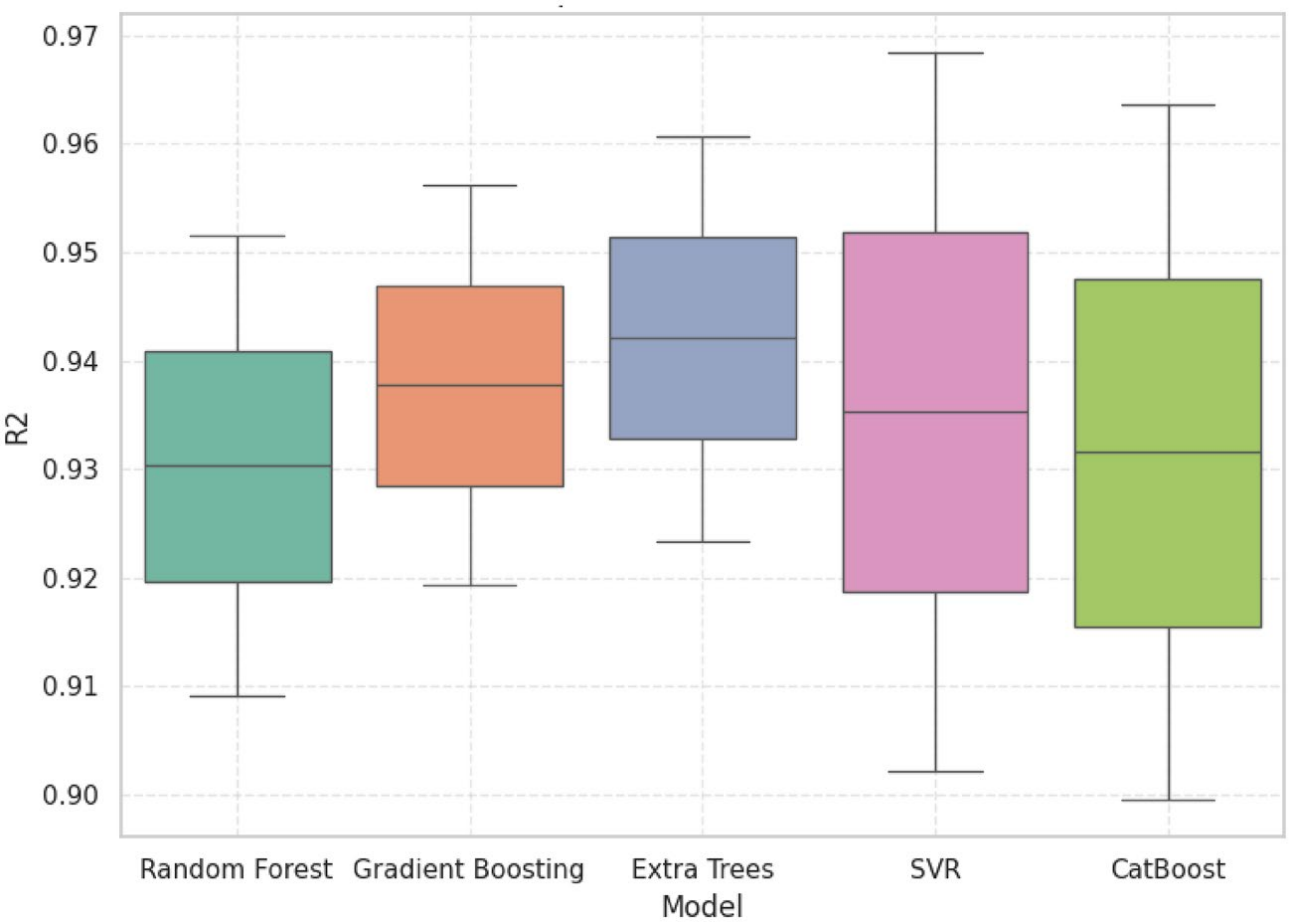
Comparative model performance R^2^ scores.

Figure 16 indicates that all models were well predictive (R^2^ > 0.90) though Gradient Boosting produced one of the highest median R^2^ = − 0.94 − 0.95 with a small interquartile range, indicating the accuracy as well as the stability of the model. ET and CB also achieved high R^2^ values although their variability is broader indicating less generalization ability. Its stable behavior under conditions contributes to the fact that GB is better at the modeling of complex system behavior without overfitting.

**Fig. 17:**
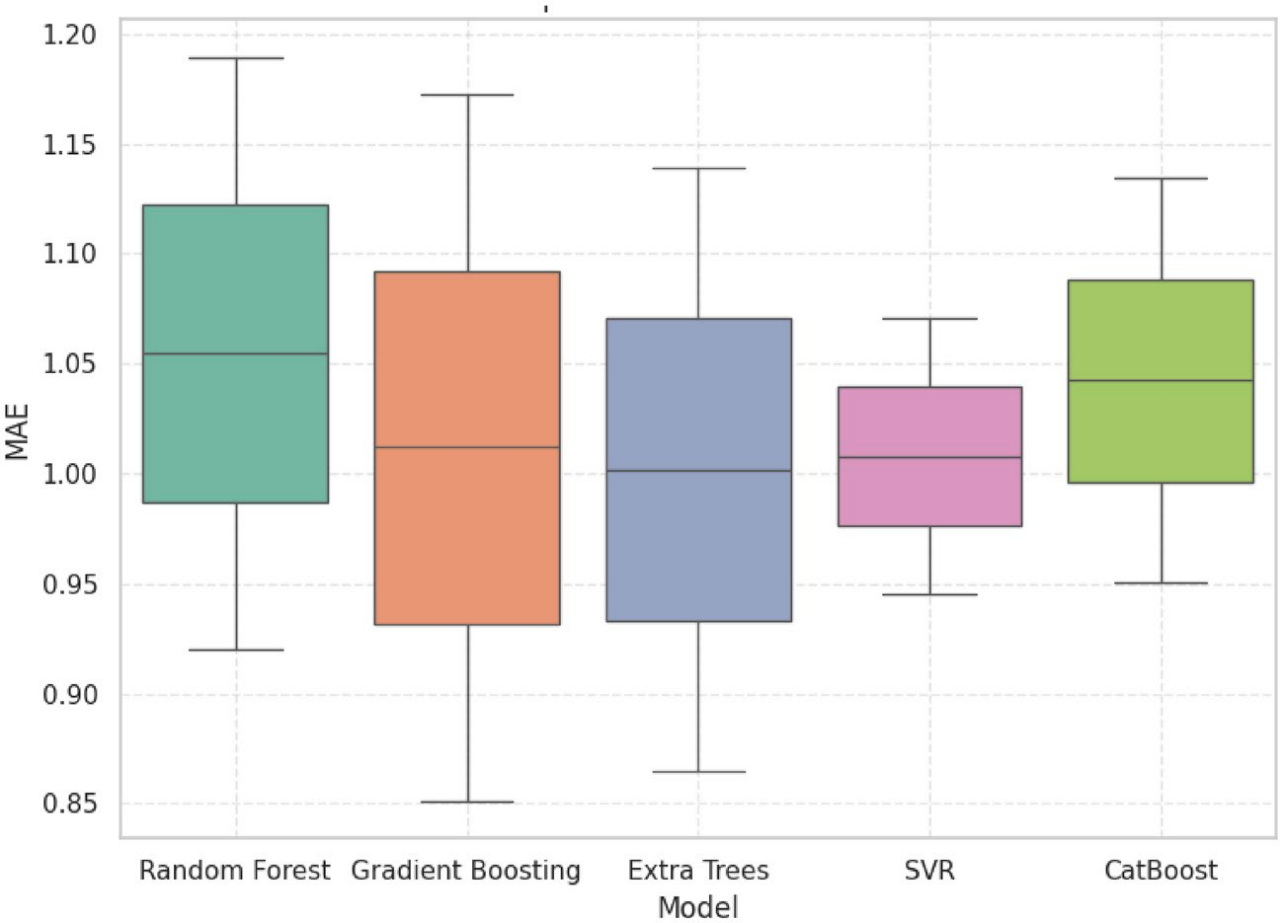
Error magnitude analysis MAE distribution.

The MAE analysis proves that GB can reduce the deviation of predictions and its median error =1.01 is low with a small error spread. Although ET had a slightly lower median MAE, its more distributed distribution is less stable. Small dispersion but poor accuracy was exhibited by SVR, which restricts its practical use as depicted in Fig. 17. GB therefore offers optimal trade-off between minimization of errors and reliability which makes it more resistant to deployment.

**Fig. 18:**
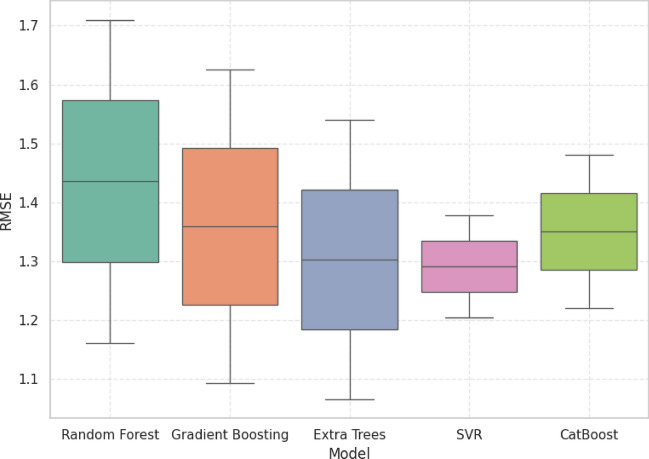
Error magnitude analysis RMSE distribution.

Based on Fig. 18, the RMSE findings indicate that GB is very robust and the median of RMSE is approximately 1.35, and the dispersion is consistent in all the predictions. Competitiveness of ET and CB at the expense of wider spreads, indicating the occasional gross errors, whereas SVR was very compact but not very accurate. Through the low RMSE and the stability of distribution, GB will prove to be more reliable in practice.

**Fig. 19:**
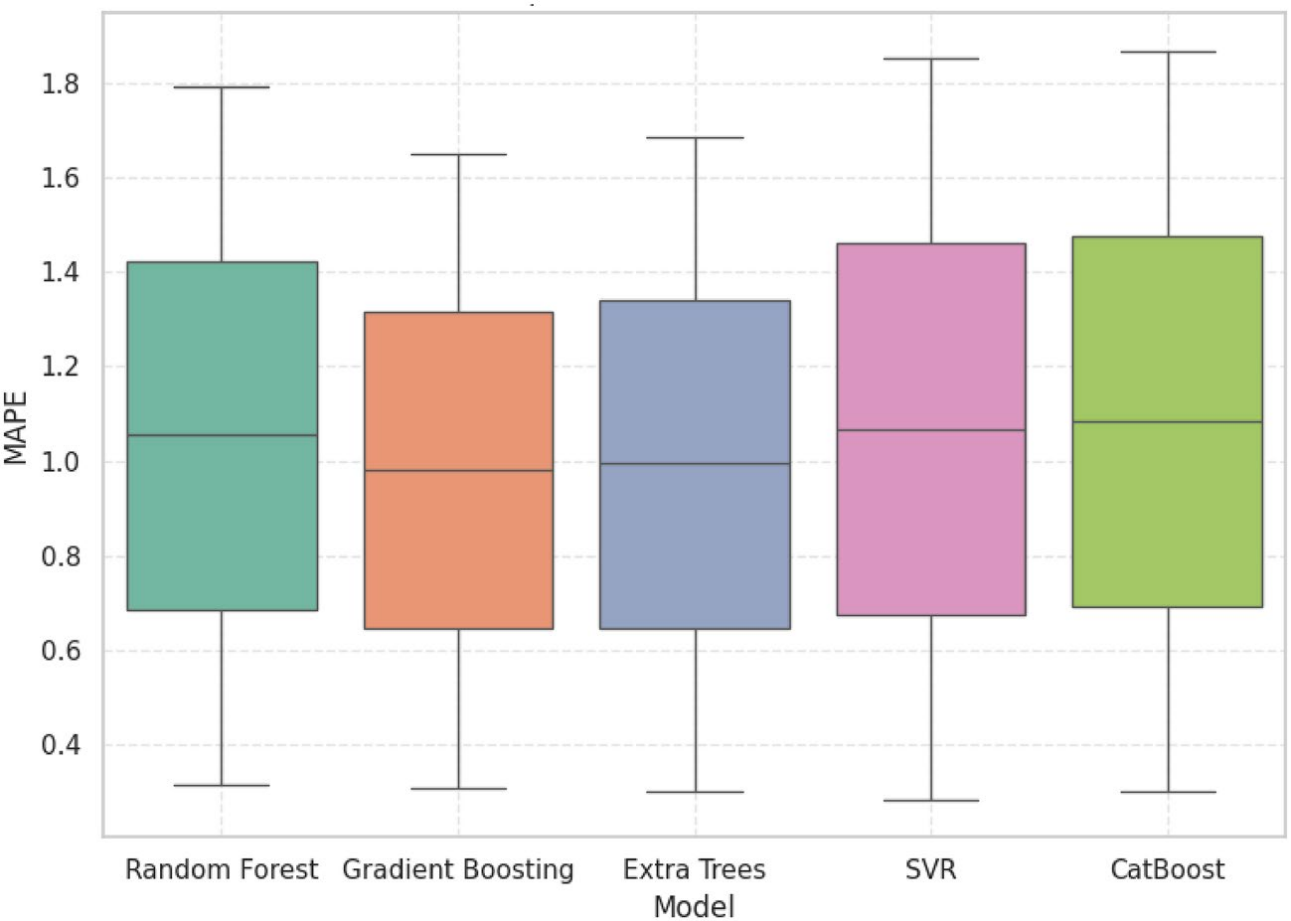
Relative error assessment MAPE distribution.

Figure 19 discusses the MAPE distribution demonstrates that GB is strong at controlling percentage-based errors which has kept the median values within the range of sub1.1 with a constant spread. As much as SVR had the lowest minimum error, it had greater variability in its upper quartiles showing uneven performance. ET and CB showed good results in the competition, however, the low median error and controlled variability of the Gradient Boosting makes it the most reliable model to use in precision prediction tasks.

### Statistical performance benchmarking of predictive models for grid stability analysis

The comparative analysis based on R^2^, MAE, RMSE, and MAPE show that GB has the best balanced and reliable performance of all the models. Although the individual metrics of ET and CB matched or were slightly higher than GB, their more dispersed error and weaker generalization decreased their strength. In the same way, SVR obtained narrow error distributions but poor predictive accuracy. GB on the other hand had the best accuracy with the lowest and constant error rates across all tests which reaffirm its high quality in precision and consistency. These findings have pointed out GB as the most strong and operationally feasible approach of forecasting grid linked hybrid PV system performance.

Table 5 presents 5 machine learning models (CB, ET, GB, RF, and SVR) in three important measures (R^2^, MAE and MAPE) of predictive performance. The relative analysis of the alternative regression models is summarized. GB was always found to have a better predictive ability compared to all models used. GB had a competitive R^2^ of 0.9373 which is very close to the maximum attained by ET=0.9419, in accuracy. More to the point, GB had the lowest RMSE =1.2628, which means that it is strong at reducing the existence of large deviations between actual and predicted values. On the same note, GB was the lowest in MAPE=0.9820 which confirms the correctness of the algorithm in minimizing percentage-based prediction errors and guaranteeing accurate performance in generalization. CB and SVR were moderately good in their performance and their error rates were worse than GB whereas the RF was the worst performer with relatively high error rates. All in all, these findings bequeath GB as the most productive and sustainable model to utilize the given data, which is why it is the best option to be considered in the study as a reliable predictor.

**Table 5:** Statistical performance comparison of machine learning models for predictive accuracy and error metrics.

Model	R2 mean	Median	Std	Min	Max	MAE mean	Median
Statistical summary per model							
Cat Boost	0.9316	0.9316	0.9316	0.8995	0.9636	1.0424	1.0424
Extra Trees	0.9419	0.9419	0.0265	0.9232	0.9607	1.0021	1.0021
Gradient Boosting	0.9373	0.9373	0.0266	0.9185	0.9561	1.0129	1.0129
Random Forest	0.9304	0.9304	0.0299	0.9093	0.9515	1.0525	1.0525
SVR	0.9353	0.9353	0.0469	0.9021	0.9685	1.0078	1.0078

Model	Std	Min	Max	Mean	Median	Std	Min
RSME							
Cat Boost	0.1302	0.9504	1.1345	1.3509	1.3509	0.1839	1.2208
Extra Trees	0.1935	0.8653	1.1389	1.3035	1.3035	0.3337	1.0676
Gradient Boosting	0.2257	0.8533	1.1726	1.2628	1.2628	0.3720	1.0998
Random Forest	0.1938	0.9155	1.1896	1.4350	1.4350	0.3887	1.1602
SVR	0.0889	0.9449	1.0706	1.2915	1.2915	0.1224	1.2049

Model	Max	Mean	Median	Std	Min	Max
MAPE						
Cat Boost	1.4809	1.0838	1.0838	1.1083	0.3002	1.8675
Extra Trees	1.5395	0.9941	0.9941	0.9791	0.3018	1.6864
Gradient Boosting	1.6258	0.9820	0.9820	0.9495	0.3105	1.6534
Random Forest	1.7098	1.0494	1.0494	1.0394	0.3145	1.7844
SVR	1.3780	1.0675	1.0675	1.1078	0.2842	1.8509

Beyond prediction accuracy, the models provide meaningful physical insights. Feature importance analysis shows that irradiance, DC-link voltage, and *d*_*q*_ currents are dominant predictors of both *ζ* and *S*, which is consistent with inverter based stability theory. The interpretability analysis also reveals that high-frequency variations in current magnitude correlate strongly with lower stability scores, aligning with physical expectations of weak grid oscillatory behavior. These observations reinforce the credibility of the ML predictions.

## Practical implications

The suggested correlation-weighted machine learning framework has a great operational value to smart-grid management. Dynamic voltage regulation is made possible by real time grid voltage predictions, which can be used by the operators to control the reactive-power of an inverter in a finer way and negate overvoltage or undervoltage occurrences when irradiance changes rapidly. The feature-sensitivity analysis also facilitates adaptive inverter tuning and coordinated dispatch since it determines the parameters that have the most significant impact on the stability. Moreover, the projection of stability also delivers to utilities an early warning system to plan reliability, allowing them to prioritize whose feeders or PV units to work on to have lower dynamic margins. The interpretation-friendly structure also enables regulatory acceptance and enhances the integration of AI supported decision-support systems in the renewable-based distribution networks due to the interpretable structure.

On the whole, this framework connects information-based intelligence to feasible grid operation approach that offers a scalable platform to predictive control, grid enhancement, and informed decision making in hybrid PV power grid. The implementation of the suggested correlation weighted Gradient Boosting framework into the hybrid PV system operations will bring quantifiable economic advantages through limiting the losses associated with the instability and enhancing the potential asset use. Real time prediction of grid voltage and stability allow the early detection of undervoltage and overvoltage conditions to avoid cases of inverter tripping tendencies which normally lead to curtailment of power and revenue. A small 1 to 2% cutback can result in considerable yearly energy savings of medium scale PV facilities. Enhanced stability prediction is also useful when it comes to more efficient reactive power scheduling, which decreases reliance on external voltage support systems and decreases Operational Expenditure (OPEX). Moreover, predictive stability scoring can help utilities optimize their maintenance cycle: by estimating the time of poor dynamic performance, the operators can adapt to condition based maintenance and abandon fixed interval maintenance. To save on maintenance expenses, this transition can save 12-18 percent as per the conventional grid asset management standards. Also, lessening the voltage deviations and ramp rate regressions lengthen the inverter life and hence reduces the long-term Capital Expenditure (CAPEX). Altogether, the suggested predictive framework improves the reliability of the system as well as its cost effectiveness, which makes it an effective means of the grid environment that is rich in renewable resources.

A comparative examination reveals clear performance distinctions. Gradient Boosting outperforms RF and ET due to its sequential residual-learning mechanism, which captures subtle nonlinear interactions between PV operating conditions and inverter dynamics. CatBoost performs competitively but is slightly less stable under high-dimensional feature interactions. SVR underperforms in transient regions because its kernel mapping is less adaptive to abrupt system nonlinearities. These findings align with the physical interpretation of the hybrid system, where nonlinear PV behavior, load sensitivity, and DC-link dynamics require models that can learn complex hierarchical patterns—an advantage naturally provided by boosting-based methods.

## Conclusion

This study presented a correlation-guided machine learning framework for predicting grid voltage deviation and dynamic stability in hybrid photovoltaic systems. Five regression models, Random Forest, Extra Trees, Support Vector Regression, CatBoost, and Gradient Boosting were evaluated under a unified setup using simulation data generated from a physics-based DAE model. Among them, Gradient Boosting delivered the most reliable performance, achieving the highest accuracy and lowest error metrics for both voltage prediction (R2 = 0.9785, MAPE = 0.25%) and stability forecasting (R2 = 0.9300, MAPE = 1.45%). he proposed framework integrates correlation-weighted feature engineering with ensemble learning, improving both predictive accuracy and interpretability. By emphasizing dominant physical drivers such as grid current, irradiance, and load demand, the model demonstrates strong robustness under variable environmental and operational conditions. These results highlight the practical value of data-driven stability prediction for real-time voltage regulation, inverter tuning, and reliability assessment in renewable-rich distribution networks. Overall, the findings confirm that combining correlation-driven feature weighting with advanced ensemble regressors enhances stability forecasting capability in hybrid PV systems. Future work will extend the framework to real-time adaptive control, multi-source renewable testbeds (PV-wind), and larger-scale smart grid applications to support predictive and resilient grid operation.

## Data Availability

Data will be made available on request.
